# Estimating the associations between women’s maltreatment in childhood and inflammatory biomarker levels prior to and during pregnancy

**DOI:** 10.1371/journal.pone.0331905

**Published:** 2025-09-08

**Authors:** Bohao Wu, Chirag M. Vyas, Adrián A. Medina, Natalie Slopen, Shruthi Mahalingaiah, Jorge E. Chavarro, Rajarshi Mukherjee, Marc Weisskopf, Andrea L. Roberts

**Affiliations:** 1 Department of Environmental Health, Harvard T.H. Chan School of Public Health, Boston, Massachusetts, United States; 2 Department of Psychiatry, Massachusetts General Hospital, Boston, Massachusetts, United States; 3 Department of Social and Behavioral Sciences, Harvard T.H. Chan School of Public Health, Boston, Massachusetts, United States; 4 McLean Imaging Center, McLean Hospital, Belmont, Massachusetts, United States; 5 Department of Nutrition, Harvard T.H. Chan School of Public Health, Boston, Massachusetts, United States; 6 Department of Epidemiology, Harvard T.H. Chan School of Public Health, Boston, Massachusetts, United States; 7 Channing Division of Network Medicine, Department of Medicine, Brigham and Women’s Hospital, Harvard Medical School, Boston, Massachusetts, United States; 8 Department of Biostatistics, Harvard T.H. Chan School of Public Health, Boston, Massachusetts, United States; The Hong Kong Polytechnic University, HONG KONG

## Abstract

**Background:**

Maternal childhood maltreatment has been associated with higher risk of adverse neurodevelopment in offspring. Chronic systemic inflammation has been associated with childhood maltreatment and has been identified as a gestational risk factor for adverse neurodevelopment in offspring. Thus, inflammation may be a mechanism by which maternal exposure to maltreatment affects offspring neurodevelopment. To estimate associations between women’s childhood maltreatment and four inflammatory biomarkers, C-reactive protein (CRP), interleukin-6 (IL-6), tumor necrosis factor-alpha receptor 2 (TNF-R2), and interferon gamma (IFN-γ), among women prior to or during pregnancy.

**Methods:**

A sub-study of the Nurses’ Health Study 3, a prospective cohort, included 329 women, 204 who were contemplating pregnancy and 124 who were pregnant. Approximately 90% of participants were non-Hispanic White and over 60% had a Master’s degree or higher. Maltreatment was assessed using the validated 28-item Childhood Trauma Questionnaire. Associations were calculated using generalized estimating equations.

**Results:**

Fifty participants (15.2%) were not exposed abuse (n = 28 contemplating pregnancy; n = 22 pregnant) and 81 participants (24.6%) were exposed to moderate or severe abuse (n = 55 contemplating pregnancy; n = 26 pregnant). Childhood maltreatment was not associated with the four inflammatory biomarkers either among participants contemplating pregnancy or among pregnant participants.

**Conclusions:**

No statistically significant associations were identified between childhood maltreatment and selected inflammatory biomarkers in this sample of well-educated, primarily non-Hispanic White women. These findings should be interpreted with caution given the limited statistical power and measurement variability. Further investigation of these associations in more vulnerable populations might enhance our understanding of biological mechanisms linking maternal childhood abuse to adverse neurodevelopment in offspring.

## Introduction

Children born to women who experienced childhood maltreatment, compared to those who did not, are at an increased risk for atypical neurodevelopment, including higher risk of internalizing behaviors [1,2], externalizing behaviors [3–5], social difficulties [3,6], and being diagnosed with autism spectrum disorder (ASD) [7,8] and attention-deficit hyperactivity disorder (ADHD) [9]. Chronic systemic inflammation, including C-reactive protein (CRP), interleukin-6 (IL-6), and tumor necrosis factor-alpha (TNF-α), has been associated with childhood maltreatment, [10–13] and systemic inflammation has been identified as a gestational risk factor for adverse neurodevelopment in offspring. High gestational concentrations of CRP, IL-6, TNF-α, and interferon-γ (IFN-γ) have been associated with greater risk of ASD or ADHD in offspring [13–16] as well as other adverse outcomes, such as adiposity and cardiometabolic health [17,18].

Pregnancy represents a critical period for the potential transmission of adverse health outcomes from mothers to children [13]. During pregnancy, women’s inflammatory responses are heightened, and serum inflammatory biomarkers are elevated compared to non-pregnant normal ranges, including CRP, IL-1β, IL-6, TNF-α, and IFN-γ [19–21]. However, excessively elevated levels of these biomarkers may negatively impact the neurodevelopment of offspring. Women who experienced childhood maltreatment, versus those who did not, may have greater inflammatory biomarker concentrations during pregnancy.

Recent systematic reviews have highlighted an association between maternal childhood abuse and increased levels of CRP, IL-6, and TNF-α [10,22,23]. However, few studies have been comprised predominantly of women of childbearing age; thus, their relevance to gestational risk is unclear. Regarding specific biomarkers, studies of middle-aged men and women have found associations of childhood maltreatment with elevated CRP [24], as well as no association with CRP concentration [11,25,26]. A single study of younger participants (age 32 years) found childhood maltreatment associated with greater CRP [27]. Two large studies of IL-6 have found childhood maltreatment associated with increased IL-6 concentration; however, the median age of participants in these studies was 57 and 54 years [24,28]. Many studies investigating the association of childhood maltreatment with TNF-α concentrations have enrolled participants from selected groups, such as those with schizophrenia, bipolar disorder, or major depressive disorder [23], or caregivers of persons with dementia [29] with both positive [11] and null associations [29].

Limited studies of child maltreatment and inflammation have included pregnant women. Two studies of pregnant women in the same university-affiliated clinic found childhood maltreatment associated with increased levels of CRP [30,31]. Other samples involving pregnant women, including more diverse samples (60% of non-White, [32], 40% of non-White, [33]), women with low income [34] and adolescents [35] have not found an association of maltreatment with CRP. In pregnant women, IL-6 concentrations were not found to differ by experience of childhood maltreatment [31,35,36]. Research on the relationship between childhood maltreatment and TNF-α or IFN-γ levels during pregnancy is scarce and inconclusive. Two small studies found no association of childhood maltreatment with TNF-α levels [31,37]. No studies have examined the association of childhood maltreatment with IFN-γ levels during pregnancy. Thus, the relationship between maternal childhood maltreatment and inflammatory biomarker levels during pregnancy remains poorly characterized, and even fewer studies have explored the associations among women contemplating pregnancy. Therefore, current understanding of how maternal childhood maltreatment is associated with inflammatory biomarker concentrations before or during pregnancy is still unclear. Estimating this association can help better illustrate the biological pathway between maternal childhood maltreatment and neurodevelopment in their offspring.

To better understand the association between childhood maltreatment and systemic inflammation both in women of childbearing age and during pregnancy itself, we recruited women contemplating pregnancy (either planning to become pregnant in the next year or actively trying) and pregnant women who were participants in an ongoing longitudinal cohort, the Nurses’ Health Study 3 (NHS3). We hypothesized that childhood maltreatment would be associated with increased levels of the inflammatory biomarkers CRP, IL-6, TNF-α, and IFN-γ.

## Materials and methods

### Study design and population

Starting in 2010, the NHS3 has enrolled female and male nurses and nursing students in the United States and Canada born on or after January 1, 1965 [38]. As of January 2023, 50,150 people had consented to join the study and 36,010 people had completed at least one follow-up questionnaire. Approximately every 6 months, participants are followed with questionnaires regarding lifestyle, reproductive, and health characteristics.

On October 20^th^ 2020, the Maternal Health, Maternal Biology (MHMB) sub-study of NHS3 was launched to examine associations of childhood maltreatment with biomarkers before and during pregnancy, and ended on February 24^th^ 2025. On each NHS3 study questionnaire, women are asked “are you currently pregnant?” and, if not, “are you actively trying to become pregnant or do you think that you may become pregnant at some point within the next year?” Women who were trying or thought they would become pregnant (henceforth termed “contemplating pregnancy”) or who were currently pregnant and at less than 24 weeks gestation were asked if they were willing to donate blood or hair samples, and if so, were invited to participant in the sub-study.

Women who consented to participate were asked to fill out a questionnaire querying experiences of childhood maltreatment. For biosample collection, women contemplating pregnancy were immediately shipped a collection kit. Women who were currently pregnant were shipped a collection kit no earlier than pregnancy week 14. Participants had blood drawn at a nearby Quest Diagnostics or via an at-home blood draw service. Refrigerated blood samples were shipped to the Channing Laboratory of Mass General Brigham. On receipt, samples were centrifuged, aliquoted, and frozen at −80°. Frozen samples were sent in a single shipment to the Rifai Laboratory of Boston Children’s Hospital for biomarker assay.

**Fig 1:**
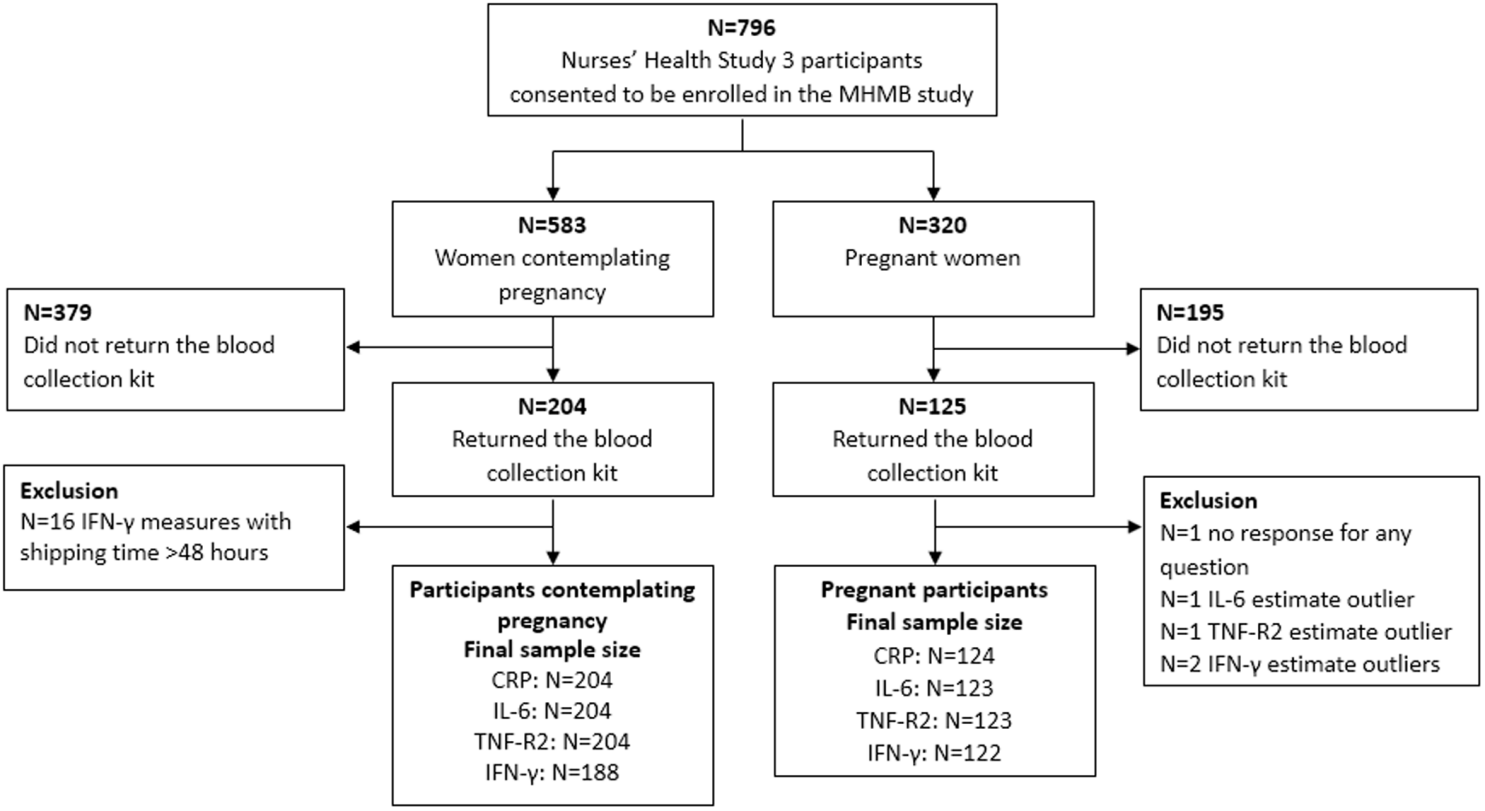
Flowchart of Maternal Health, Maternal Biology study enrollment.

As of January 2023, 796 participants completed the sub-study baseline questionnaire, including 583 women contemplating pregnancy and 320 pregnant women. Prior studies of pregnant women have also had low or moderate blood sample donation percentages from participants (40.9% [35]; 26.2% [34]). Among these participants, 329 women (204 women contemplating pregnancy and 125 pregnant women; 29 women participated both before and during pregnancy) returned a blood collection kit (Fig 1). Among women contemplating pregnancy, participants who did not return blood kits were of similar age, education attainment, and smoking history as women who did return kits. They were more likely to be obese. Among pregnant women, participants who did not return blood kits were also of similar age and education attainments as women who did return kits, but they were less likely to be overweight or obese, and more likely to have smoking history (S1 and S2 Tables). One participant (0.3%) did not respond to the blood draw questionnaire and was excluded.

Informed written consent was provided by participants at sub-study enrolled. Study protocols were approved by the Institutional Review Board of Partners HealthCare (protocol #2006P000473, initial approval date: December 27, 2018).

### Measures

#### Childhood maltreatment.

The Childhood Trauma Questionnaire (CTQ) [39,40], a commonly used childhood trauma assessment method in both research and clinical contexts, consists of 28 items measuring 5 types of childhood maltreatment, including emotional abuse, physical abuse, sexual abuse, emotional neglect, and physical neglect. The 28 items all start with the phrase “When I was growing up, during my first 18 years of life, …” Items query, for example, not having enough to eat, whether the respondent had someone to take care of and protect me, and whether they were called names, such as stupid, lazy, or ugly. Response options were ‘never true’ (score 1), ‘rarely true’ (score 2), ‘sometimes true’ (score 3), ‘often true’ (score 4) and ‘very often true’ (score 5). Responses to positive items, such as whether they felt loved, were made to feel important, and were looked out for, were reverse coded, from ‘never true’ (score 5) to ‘very often true’ (score 1). We calculated a CTQ total score by summing responses to all questions. Using the original cutoff score of >36 [40], 24.6% of study participants were classified as having experienced childhood maltreatment. To exclude participants who had any adverse experiences from the reference group, we considered participants having a score of 25 (the lowest possible score) as having not experienced any maltreatment. Participants having a score greater than 25 were classified by approximate tertile into three groups: very low (score 26–29), low (score 30–36), or moderate or high maltreatment (score >36). We also examined the CTQ score as a continuous variable.

#### Biomarker concentrations.

High sensitivity CRP was measured using an immunoturbidimetric assay on the Roche Cobas 6000 system (Roche Diagnostics – Indianapolis, IN), with reagents and calibrators from Roche. This high-sensitivity assay has a limit of detection (LOD) of hsCRP at 0.1 mg/L. The intra-assay coefficient of variation (CV) was 4.3%. IL-6 was measured using an ultra-sensitive Enzyme-Linked Immunosorbent Assay (ELISA, R & D Systems, Minneapolis, MN). This assay has a LOD of IL-6 at 0.031 pg/mL. The intra-assay CV was 8.6%. TNF-R2 was measured using an ELISA assay (R & D Systems, Minneapolis, MN). This assay has a LOD of TNF-R2 at 2.3 pg/mL. The intra-assay CV was 4.6%. IFN-γ was measured using an ultra-sensitivity ELISA assay (R & D Systems, Minneapolis, MN). This assay has a LOD of IFN-γ at 0.078 pg/mL. The intra-assay CV was 20.1%.

Biomarker concentrations were examined for normality and to detect outliers. Due to the non-normality of the biomarker values, log transformation of CRP values, reciprocal transformation of the square root of IL-6 values, reciprocal transformation of TNF-R2 values, and reciprocal transformation of the square root of IFN-γ values were performed based on the Box-Cox transformation method. Measures that were 3 standard deviations greater than or less than the mean of the transformed biomarker values were considered outliers [41]. Among the 204 participants contemplating pregnancy, no participant had levels of CRP, IL-6, TNF-R2, or IFN-γ that were considered outliers. Among the 124 pregnant participants, no CRP, 1 (0.8%) IL-6, 1 (0.8%) TNF-a, and 2 (1.6%) IFN-γ measures were considered outliers (Fig 1).

Some biomarkers deteriorate when refrigerated for longer than 48 hours. Therefore, we examined the association of biomarker concentration with shipping time. Using the Wilcoxon two-sample test, we examined whether biomarker measures were significantly higher or lower when shipping time was greater than versus less than 48 hours. Based on these analyses, we excluded 16 IFN-γ measurements (7.8%).

#### Demographics and pregnancy characteristics.

Participants’ date of birth, racial identity (White, Black, Asian, Native Hawaiian or other Pacific Islander, American Indian or Alaska Native, and other ancestry), ethnicity (Hispanic, non-Hispanic), and height were self-reported on the NHS3 baseline questionnaire. Gestational age, weight, smoking, e-cigarette usage, marijuana usage, and current medication usage were self-reported at the time of blood draw. Body mass index (BMI, kg/m^2^) at the time of blood draw was classified as underweight, healthy weight, overweight, or obese using World Health Organization standards [42]. Educational attainment and history of cancer, asthma, cardiovascular diseases, auto-immune diseases, and eczema were from NHS3 follow-up questionnaires. Education attainment was classified into four groups: less than bachelor’s degree, bachelor’s degree or registered nurse, master degree, or doctorate degree. Nurses characterized as nursing students, having associates’ degree or diploma in nursing, or being licensed practical nurse were combined in the ‘less than bachelor’s degree’ group. At the time of the blood draw, hours since the most recent food or beverage consumption (other than water) was queried. Hours since waking was calculated from the time of morning awakening (“when your eyes first opened”) and the time of the blood draw.

#### Missing values.

Values below the LOD for biomarkers (CRP: n = 9, 2.7%) were imputed as the corresponding LOD for each biomarker divided by the square root of 2 [43]. A single missing value for the CTQ score (n = 1, 0.3%) was imputed using similar questions from the main NHS3 following-up questionnaires (see S3 Table in the Supplementary Files). Missing dates of blood draw were replaced with the date the blood sample was received at the Channing Laboratory (n = 7, 2.1%). Missing values for gestational age at the time of blood draw were calculated from the gestational week reported when participants were invited to the MHMB study and the time between this date and the date of the blood draw (n = 3, 2.4%). Missing values for weight (n = 3, 0.9%) were drawn from the most recent NHS3 questionnaire before the blood draw.

### Statistical analysis

We examined demographic and pregnancy-related characteristics by child maltreatment category (none, very low, low, and moderate or high maltreatment). Covariates included in the multivariable models were selected based on previous literature as either nuisance variables (i.e., variables that would affect biomarker levels and were not likely caused by childhood maltreatment such as week of gestation), or factors that might be on a pathway between maltreatment and biomarker level, such as BMI at the time of blood draw [20,31,35,44,45].

To estimate the association of childhood maltreatment with inflammatory biomarker concentration while accounting for nuisance variables, we fit a model with biomarker concentration as the dependent variable and CTQ score (as categorical variable: none, very low, low, and moderate or high maltreatment; as continuous variable: CTQ score), age, gestational age, racial identity, hours since waking, and hours since most recent food or drink consumption as the independent variables, separately for each biomarker. To identify the proportion of inflammation associated with maltreatment that is due to health-related factors associated with maltreatment, such as BMI and substance use, we fit a second model for each biomarker further adjusted for factors that may be on the path between childhood maltreatment and inflammation, namely, BMI, education attainment, smoking, e-cigarette, marijuana, and medication usage.

Finally, to examine overall inflammation using all four biomarkers jointly, we conducted principal component analysis (PCA) and calculated the first principal component of inflammation for each participant.

We conducted three sensitivity analyses. First, to examine risk of very high concentrations of inflammatory biomarkers, we created a binary variable for each biomarker, indicating highest 10% versus lowest 90% of biomarker concentration. We then fit logistic regression models using this indicator as the dependent variable. Second, we included outliers in the analyses. Third, to enhance statistical power, we conducted analyses including women contemplating pregnancy and pregnant women in a single model and included a variable indicating participants’ pregnancy status.

Generalized estimating equations with a normal distribution and identity link were used since, for quality control purposes, 33 participants’ biomarker levels were measured twice. An unstructured covariance matrix was used for modeling the correlation between repeated measures within participants, since it had the best fit [46]. For all statistical analyses, p-value <0.05 was considered statistically significant. Analyses were performed using SAS version 9.4 (SAS Institute, Inc.).

## Results

### Sample characteristics

Characteristics of study participants who both completed questionnaires and returned blood kits are presented in Table 1 (participants contemplating pregnancy) and Table 2 (pregnant participants). Among participants contemplating pregnancy, 13.7% (n = 28) had not been exposed to any maltreatment, 28.9% (n = 59) had been exposed to very low, 30.4% (n = 62) to low, and 27.0% (n = 55) to moderate or high maltreatment. The median of age at blood kit return 

**Table 1:** Characteristics of study participants contemplating pregnancy in the total sample (n = 204) and by severity levels of childhood maltreatment, Nurses’ Health Study 3.

Variable		Childhood maltreatment									
		No maltreatment (score 25, n = 28, 13.7%)		Very low maltreatment (score 26–29, n = 59, 28.9%)		Low maltreatment (score 30–36, n = 62, 30.4%)		Moderate or severe maltreatment (score >36, n = 55, 27.0%)		Total (n = 204)	
		N or Median	% or Q1-Q3	N or Median	% or Q1-Q3	N or Median	% or Q1-Q3	N or Median	% or Q1-Q3	N or Median	% or Q1-Q3
**Biomarker values**	HsCRP, mg/L	1.01	0.36-2.68	0.58	0.34-1.54	1.30	0.54-3.92	0.59	0.30-1.49	0.81	0.36-2.45
	IL-6, pg/mL	0.58	0.41-0.87	0.54	0.38-0.79	0.66	0.42-1.12	0.54	0.39-1.00	0.56	0.40-0.92
	TNF-R2, pg/mL	2093.65	1695.60-2586.40	1884.40	1624.20-2209.90	2089.40	1779.80-2365.80	2002.90	1682.90-2246.10	2012.75	1694.00-2365.55
	IFN-γ, pg/mL	0.53	0.43-0.69	0.54	0.41-0.86	0.48	0.36-0.77	0.46	0.39-0.62	0.49	0.38-0.73
**Age, yrs**	Age entered NHS3	26.4	24.0-29.1	26.6	24.5-29.5	26.5	24.4-29.5	26.5	24.7-29.2	26.5	24.5-29.3
	Age had the blood test	35.0	33.3-37.1	33.9	31.9-38.6	33.8	31.8-37.4	35.1	32.4-38.1	34.5	32.1-37.7
**Race**	White	27	96.4	53	89.8	53	85.5	47	85.5	180	88.2
	Non-White	1	3.6	6	10.2	9	14.5	8	14.6	24	11.8
**Ethnicity**	Hispanic	2	7.1	4	6.9	3	4.9	3	5.6	12	6.0
	Non-Hispanic	26	92.9	54	93.1	58	95.1	51	94.4	189	94.0
**Education**	Bachelor’s degree or RN, Registered Nurse	12	42.9	29	49.1	17	27.4	23	41.8	81	39.7
	Master’s degree	11	39.3	27	45.8	34	54.8	27	49.1	99	48.5
	Doctoral degree	5	17.9	3	5.1	11	17.7	5	9.1	24	11.8
**BMI**	Underweight (<18.5 kg/m^2^)	2	7.1	1	1.7	2	3.2	1	1.8	6	2.9
	Healthy weight (18.5–24.9 kg/m^2^)	15	53.6	36	61.0	27	43.6	23	41.8	101	49.5
	Overweight (25.0–29.9 kg/m^2^)	9	32.1	14	23.7	16	25.8	23	41.8	62	30.4
	Obese (>29.9 kg/m^2^)	2	7.1	8	13.6	17	27.4	8	14.6	35	17.2
**Smoking**	Never	25	89.3	51	86.4	53	85.5	43	78.2	172	84.3
	Ever smoked	3	10.7	8	13.6	9	14.5	12	21.8	32	15.7
**E-cigarette**	Never	27	96.4	56	94.9	60	96.8	47	85.4	190	93.1
	Ever used	1	3.6	3	5.1	2	3.2	8	14.5	14	6.9
**CBD**	Never	28	100	52	88.1	56	90.3	48	87.3	184	90.2
	Ever used	0	0	7	11.9	6	9.6	7	12.7	20	9.8
**Marijuana**	Never	10	35.7	25	42.4	22	35.5	17	30.9	74	36.3
	Ever used	18	64.3	34	57.6	40	64.5	38	69.1	130	63.7
**Medication**	Antidepressants	4	14.3	12	20.3	15	24.2	18	32.7	49	24.0
	Antibiotics	0	0	1	1.7	4	6.5	2	3.6	7	3.4
	Aspirin products	2	7.1	2	3.4	1	1.6	4	7.3	9	4.4
	DHEA	0	0	0	0	0	0	1	1.8	1	0.5
	Estrogen for fertility	0	0	1	1.7	3	4.8	3	5.5	7	3.4
	Hormonal contraception	6	21.4	9	15.3	8	12.9	7	12.7	30	14.7
	Ibuprofen	2	7.1	3	5.1	7	11.3	9	16.4	21	10.3
	Thyroid medications	3	10.7	7	11.9	6	9.7	7	12.7	23	11.3

HsCRP, high-sensitive C-reactive protein. IL-6, interleukin-6. TNF-R2, tumor necrosis factor-alpha receptor 2. IFN-γ, interferon-γ.

a. Childhood maltreatment was measured by the 28-item Childhood Trauma Questionnaire.

b. 29 participants took the blood test both prior to and during pregnancy.

c. Percentages may not add up to 100% due to rounding.

d. Biomarker values by CTQ category see S1 Fig.

**Table 2:** Characteristics of study pregnant participants in the total sample (n = 124) and by severity levels of childhood maltreatment, Nurses’ Health Study 3.

Variable		Childhood maltreatment									
		No maltreatment (score 25, n = 22, 17.7%)		Very low maltreatment (score 26–29, n = 41, 33.1%)		Low maltreatment (score 30–36, n = 35, 28.2%)		Moderate or severe maltreatment (score >36, n = 26, 21.0%)		Total (n = 124)	
		N or Median	% or Q1-Q3	N or Median	% or Q1-Q3	N or Median	% or Q1-Q3	N or Median	% or Q1-Q3	N or Median	% or Q1-Q3
**Biomarker values**	HsCRP, mg/L	3.03	1.66-5.41	3.75	1.79-8.09	3.36	1.54-8.25	3.58	2.41-6.82	3.42	1.79-7.69
	IL-6, pg/mL	0.63	0.41-0.71	0.63	0.43-0.97	0.64	0.52-1.03	0.57	0.45-0.81	0.62	0.46-0.91
	TNF-R2, pg/mL	2440.65	2143.30-2810.20	2457.50	2144.10-2862.50	2560.60	2208.30-2859.50	2530.05	2175.50-3159.70	2484.70	2167.15-2894.95
	IFN-γ, pg/mL	0.46	0.42-0.64	0.45	0.31-0.76	0.45	0.38-0.64	0.44	0.37-0.78	0.45	0.37-0.67
**Age**	Age entered NHS3	27.3	24.4-29.5	25.8	24.2-27.6	25.7	23.4-29.0	27.2	25.0-29.2	26.2	24.3-28.8
	Age had the blood test	34.5	33.2-37.0	33.4	31.0-35.4	34.3	31.9-36.2	33.8	32.6-36.3	33.8	32.2-36.1
**Race**	White	22	100	39	95.1	33	94.3	24	92.3	118	95.2
	Non-White	0	0	2	4.9	2	5.7	2	7.7	6	4.8
**Ethnicity**	Hispanic	1	4.6	1	2.4	5	14.3	2	7.7	9	7.3
	Non-Hispanic	21	95.5	40	97.6	30	85.7	24	92.3	115	92.7
**Education**	Under bachelor’s degree	0	0	0	0	1	2.9	0	0	1	0.8
	Bachelor’s degree/ RN, Registered Nurse	7	31.8	13	31.7	8	22.9	14	53.9	42	33.9
	Master degree	9	40.9	23	56.1	19	54.3	10	38.5	61	49.2
	Doctorate degree	6	27.3	5	12.2	7	20.0	2	7.7	20	16.1
**BMI**	Underweight (<18.5 kg/m^2^)	0	0	2	4.9	0	0	1	3.9	3	2.4
	Healthy weight (18.5–24.9 kg/m^2^)	10	45.4	15	36.6	17	48.6	10	38.5	52	41.9
	Overweight (25.0–29.9 kg/m^2^)	8	36.4	17	41.5	8	22.9	7	26.9	40	32.3
	Obese (>29.9 kg/m^2^)	4	18.2	7	17.1	10	28.6	8	30.8	29	23.4
**Gestational age**	9-12 weeks	10	45.5	10	24.4	7	20	5	19.2	32	25.8
	13-28 weeks	9	40.9	28	68.3	25	71.4	18	69.2	80	64.5
	29-33 weeks	3	13.6	3	7.3	3	8.6	3	11.5	12	9.7
	Median, Q1-Q3	13.5	12.0-23.0	18.0	13.0-25.0	17.0	14.0-25.0	17.5	13.0-23.0	17.0	12.0-25.0
**Smoking**	Never	21	95.5	39	95.1	33	94.3	22	84.6	115	92.7
	Ever smoked	1	4.6	2	4.9	2	5.7	4	15.4	9	7.3
**E-cigarette**	Never	22	100	39	95.1	34	97.1	24	92.3	119	96.0
	Ever used	0	0	2	4.9	1	2.9	2	7.7	5	4.0
**Marijuana**	Never	9	59.1	24	58.5	16	45.7	8	30.8	57	46.0
	Ever used	13	40.9	17	41.5	19	54.3	18	69.2	67	54.0
**Medication**	Antidepressants	0	0	2	4.9	7	20.0	5	19.2	14	11.3
	Aspirin products	6	27.3	7	17.1	10	28.6	6	23.1	29	23.4
	Thyroid medications	2	9.1	4	9.8	4	11.4	4	15.4	14	11.3

HsCRP, high-sensitive C-reactive protein. IL-6, interleukin-6. TNF-R2, tumor necrosis factor-alpha receptor 2. IFN-γ, interferon-γ.

a. Childhood maltreatment was measured by the 28-item Childhood Trauma Questionnaire.

b. 29 participants took the blood test both prior to and during pregnancy.

c. Percentages may not add up to 100% due to rounding.

d. Biomarker values by CTQ category see S2 Fig.

was 34.5 years (interquartile range [IQR]=32.1–37.7, range = 25.7–50.1); most participants (88.2%) were White and 60.3% had a master’s or doctorate degree. Compared with women who did not experience childhood maltreatment, participants who experienced any maltreatment were older, had a higher prevalence of smoking, were more likely to have e-cigarette usage history, and were more likely to have medication usage including antidepressants, estrogen for fertility, and ibuprofen. Participants who had not been exposed to any childhood maltreatment, versus those who had, were more likely to be using hormonal contraception at the time of blood draw.

Among pregnant participants, 17.7% (n = 22) had not been exposed to any maltreatment, 33.1% (n = 41) had been exposed to very low, 28.2% (n = 35) to low, and 21.0% (n = 26) to moderate to high maltreatment. The median of age at the blood draw was 33.8 years (IQR = 32.2–36.1, range = 23.6–42.7). Median gestational age was 17 weeks (IQR 12–25, range 9–33). Most participants were White (95.2%) and 65.3% had a master’s or doctorate degree. Compared with pregnant women without childhood maltreatment, pregnant participants who experienced any childhood maltreatment were older, more likely to be obese at the time of blood draw, and more likely to have tobacco smoking or marijuana use history. Those exposed to any childhood maltreatment were also more likely to take antidepressant or thyroid medications compared with participants without maltreatment.

### Childhood maltreatment and inflammatory biomarkers among participants contemplating pregnancy

Among participants contemplating pregnancy (**Table 3**), in models adjusted for age, racial identity, hours since awakening, and hours since last eating or drinking, childhood maltreatment was not associated with any of the four biomarkers. In models further adjusted for BMI, educational attainment, substance use (smoking, e-cigarette, marijuana), and medication use 

**Table 3:** Associations between childhood maltreatment and biomarker estimates among participants contemplating pregnancy, N = 204, Nurses’ Health Study 3.

Biomarker	Model	CTQ categories (ref: no maltreatment)			
		Very low maltreatment	Low maltreatment	Moderate or severe maltreatment	Continuous CTQ score
		β (95% CI)	β (95% CI)	β (95% CI)	β (95% CI)
**HsCRP**	Model 1	−0.22 (−0.82, 0.39)	0.37 (−0.25, 0.99)	−0.36 (−0.99, 0.28)	−0.01 (−0.02, 0.005)
	Model 2	−0.21 (−0.73, 0.30)	0.13 (−0.44, 0.70)	−0.47 (−1.00, 0.05)	−0.01 (−0.02, 0.003)
**IL-6**	Model 1	0.03 (−0.12, 0.18)	−0.10 (−0.25, 0.04)	−0.03 (−0.18, 0.12)	−0.002 (−0.006, 0.001)
	Model 2	0.07 (−0.07, 0.22)	−0.03 (−0.17, 0.11)	0.02 (−0.12, 0.16)	−0.002 (−0.006, 0.001)
**TNF-R2**	Model 1	3.50x10^-5^ (−1.30x10^-5^, 8.30x10-^5^)	−0.27x10^-5^ (−4.92x10^-5^, 4.38x10^-5^)	2.38x10^-5^ (−2.51x10^-5^, 7.27x10^-5^)	7.43x10^-7^ (−4.65x10^-7^, 0.20x10^-5^)
	Model 2	4.37x10^-5^ (−0.13x10^-5^, 8.88x10^-5^)	1.12x10^-5^ (−3.19x10^-5^, 5.43x10^-5^)	3.38x10-^5^ (−1.25x10^-5^, 8.00x10^-5^)	8.73x10^-7^ (−2.19x10^-7^, 0.20x10^-5^)
**IFN-γ**	Model 1	−0.02 (−0.15, 0.10)	0.03 (−0.09, 0.16)	0.08 (−0.03, 0.20)	0.003 (−0.16x10^-3^, 5.41x10^-3^)
	Model 2	−0.02 (−0.14, 0.10)	0.04 (−0.08, 0.16)	0.09 (−0.03, 0.21)	**0.003 (0.20x10** ^**-3**^ **, 0.006)**
**Factor**	Model 1	−0.20 (−0.64, 0.24)	0.21 (−0.24, 0.67)	−0.22 (−0.67, 0.24)	−0.004 (−0.01, 0.004)
	Model 2	−0.29 (−0.68, 0.10)	0.003 (−0.39, 0.40)	−0.35 (−0.73, 0.03)	−0.005 (−0.01, 0.003)

HsCRP, high-sensitive C-reactive protein. IL-6, interleukin-6. TNF-R2, tumor necrosis factor-alpha receptor 2. IFN-γ, interferon-γ. SE, standard error. CTQ, childhood trauma questionnaire. Ref, reference group. CI, confidence interval.

a. Model 1: adjusted for age, race, hours after waking up, and hours since last eat or drink.

b. Model 2: adjusted for age, gestational age, race, BMI, education attainment, hours after waking up, hours since last eat or drink, ever smoked, ever used e-cigarette, ever used marijuana, and current individual medication usage (antidepressants, hormonal contraception, ibuprofen, thyroid medications).

c. Factor indicates the single factor forced from the four inflammatory biomarkers via principal component analysis (S4 Table).

d. Bold font indicates significance (P-value < 0.05).

e. Full model results see S5**-**S9 Tables.

(antidepressants, hormonal contraception, ibuprofen, thyroid medications), participants with a higher CTQ score (as the continuous measure) had a lower concentration of IFN-γ (P-value = 0.04) compared with participants having a lower CTQ score. No other associations were identified between childhood maltreatment and biomarker concentration.

To further understand our results, and as a check on the quality of the data, we compared the coefficients describing the relationship between childhood abuse and biomarker concentrations to coefficients for other covariates in the fully adjusted model. In analyses of CRP, we found CRP concentrations increased per year of age (P-value = 0.03). Women with overweight (P-value<0.001) or obesity (P-value<0.001) versus women with healthy weight, and women who had ever versus never used hormonal contraception had higher concentrations of CRP (P-value = 0.01). Women who had ever smoked (P-value = 0.04, versus women who never smoked) or women who used e-cigarettes (P-value = 0.02, versus women who never used e- cigarettes) had a lower concentration of CRP. In analyses of IL-6, obesity versus healthy weight was associated with higher concentration (P-value<0.001). In analyses of TNF-R2, hours since awakening was associated with greater concentration (P-value = 0.002). Women who ever versus never used marijuana had a lower concentration of TNF-R2 (P-value = 0.02). Obesity versus healthy weight (P-value = 0.004) and thyroid medication use (P-value = 0.03) were associated with higher TNF-R2 concentrations. In analyses of IFN-γ, participants who ever versus never used marijuana (P-value<0.05) and those who were using thyroid medications versus those who did not have higher concentrations of IFN-γ (P-value = 0.03).

In analyses with the four biomarkers combined into a single factor using principal components analysis, childhood maltreatment was not associated with this factor.

### Childhood maltreatment and inflammatory biomarkers among pregnant participants

Among pregnant participants (Table 4), in models adjusted for age, gestational age, racial identity, hours after awakening, and hours since last eating or drinking, childhood maltreatment was not associated with any of the four biomarkers. In models further adjusted for BMI, education attainment, substance usage (smoking, e-cigarette, marijuana), and medication usage (antidepressants, aspirin products, thyroid medications), no associations were identified between childhood maltreatment and biomarker concentration.

**Table 4:** Associations between childhood maltreatment and biomarker estimates among pregnant participants, N = 124, Nurses’ Health Study 3.

Biomarker	Model	CTQ categories (ref: no maltreatment)			
		Very low maltreatment	Low maltreatment	Moderate or severe maltreatment	Continuous CTQ score
		β (95% CI)	β (95% CI)	β (95% CI)	β (95% CI)
**HsCRP**	Model 1	0.23 (−0.26, 0.72)	0.22 (−0.34, 0.78)	0.36 (−0.17, 0.90)	0.01 (−0.006, 0.03)
	Model 2	0.26 (−0.16, 0.67)	0.26 (−0.26, 0.78)	0.39 (−0.10, 0.87)	0.01 (−0.001, 0.03)
**IL-6**	Model 1	−0.12 (−0.34, 0.09)	−0.11 (−0.28, 0.06)	−0.07 (−0.27, 0.13)	0.89x10^-3^ (−4.18x10^-3^, 5.95x10^-3^)
	Model 2	−0.15 (−0.36, 0.06)	−0.11 (−0.28, 0.06)	−0.09 (−0.29, 0.11)	0.69x10^-3^ (−4.20x10^-3^, 5.58x10^-3^)
**TNF-R2**	Model 1	0.95x10^-5^ (−5.20x10^-5^, 3.29x10^-5^)	0.64x10^-5^ (−4.76x10^-5^, 3.48x10^-5^)	0.82x10^-5^ (−5.35x10^-5^, 3.71x10^-5^)	−7.60x10^-7^ (−0.22x10^-5^, 6.57x10^-7^)
	Model 2	1.54x10^-5^ (−6.05x10^-5^, 2.97x10^-5^)	0.77x10^-5^ (−4.93x10^-5^, 3.40x10^-5^)	−1.27x10^-5^ (−5.93x10^-5^, 3.39x10^-5^)	−0.11x10^-5^ (−0.26x10^-5^, 0.04x10^-5^)
**IFN-γ**	Model 1	0.04 (−0.11, 0.19)	0.07 (−0.08, 0.21)	0.02 (−0.13, 0.17)	−0.003 (−0.01, 0.004)
	Model 2	0.002 (−0.15, 0.16)	0.04 (−0.10, 0.19)	−0.03 (−0.17, 0.11)	−0.002 (−0.006, 0.003)
**Factor**	Model 1	0.21 (−0.27, 0.69)	0.18 (−0.26, 0.61)	0.14 (−0.41, 0.69)	0.005 (−0.02, 0.03)
	Model 2	0.32 (−0.13, 0.78)	0.19 (−0.23, 0.61)	0.26 (−0.21, 0.73)	0.009 (−0.009, 0.03)

HsCRP, high-sensitive C-reactive protein. IL-6, interleukin-6. TNF-R2, tumor necrosis factor-alpha receptor 2. IFN-γ, interferon-γ. SE, standard error. CTQ, childhood trauma questionnaire. Ref, reference group. CI, confidence interval.

a. Model 1: adjusted for age, gestational age, race, hours after waking up, and hours since last eat or drink.

b. Model 2: adjusted for age, gestational age, race, BMI, education attainment, hours after waking up, hours since last eat or drink, ever smoked, ever used e-cigarette, ever used marijuana, and current individual medication usage (antidepressants, aspirin products, thyroid medications).

c. Factor indicates the single factor forced from the four inflammatory biomarkers via principal component analysis (S4 Table).

d. Full model results see S10**-**S14 Tables.

To further understand our results, we compared the coefficients describing the relationship between childhood abuse and biomarker concentrations to coefficients for other covariates in the fully adjusted model. In analyses of CRP, we found that overweight (P- value = 0.009) and obese (P-value<0.001) participants had a higher concentration of CRP, compared with women having a healthy weight. Compared with participants who were not using antidepressants, women who were using antidepressants had a lower concentration of CRP (P-value = 0.024). In analyses of IL-6, obese women had a higher concentration of IL-6, compared with women having a healthy weight (P-value<0.001). In analyses of TNF-R2, participants with an older gestational age (P-value<0.001) or more hours since last eating or drinking (P-value<0.001) had a higher concentration of TNF-R2. In analyses of IFN-γ, women who ever smoked versus those who never smoked had a higher concentration of IFN-γ (P-value<0.001). Underweight women (P-value = 0.001, versus women having a healthy weight) or women with more hours since last eating or drinking (P-value<0.001) had a lower concentration of IFN-γ.

In analyses with the four biomarkers combined into a single factor using principal components analysis, childhood maltreatment was not associated with this factor.

### Sensitivity analysis

Three sensitivity analyses were conducted, including (1) measuring each biomarker as binary variables (highest 10% versus lowest 90% of biomarker concentration), (2) not excluding biomarker outliers, and (3) including both women contemplating pregnancy and pregnant women in the single model. In all three analyses, no association was found between childhood maltreatment and any of the four biomarkers.

## Discussion

We found no association between women’s experience of childhood maltreatment and inflammatory biomarkers, including CRP, IL-6, TNF-R2, and IFN-γ, either prior to or during pregnancy.

Meta-analyses have indicated that individuals exposed to childhood maltreatment are more likely to have elevated CRP, IL-6, and TNF-α concentrations [10,23,47]. However, few of the studies included in these analyses were conducted among women of childbearing age. Even fewer studies have examined the association between childhood maltreatment and inflammatory biomarkers during pregnancy. Two studies conducted among participants sampled from the same academic clinic (n = 214 and n = 77) found a significant association between women’s childhood maltreatment and CRP during pregnancy [30,31]. However, no association was found in studies conducted with other pregnant samples, including a study of low-income Black, Latina, and non-Hispanic White women (n = 90) [34] or pregnant adolescents (n = 133) [35]. Our finding of no association of childhood maltreatment and IL-6 during pregnancy matches findings of prior studies [31,35,36]. Three previous studies examined the association between childhood maltreatment and TNF-R2 during pregnancy [31,37,48], and one study assessed the association of childhood hardship, consisting of low family socioeconomic status and limited healthcare access, with IFN-γ during pregnancy [48]; no association was found either with TNF-R2 or IFN-γ.

In previous studies, age, gestational age, and obesity have been associated with inflammatory biomarkers [20,31,35,36,44,45,49,50]. In our data, age was associated with CRP only among participants contemplating pregnancy. It may be that the pro-inflammatory state induced by pregnancy masked age differences [19–21]. In addition, age-associated inflammation occurs primarily after age 65 years [50]. Regarding gestational age, previous studies have found that CRP level increases before 20 weeks of gestation and then declines [45], whereas IL-6 level increases as gestational age increases [20,45]. Associations of TNF-R2 and IFN-γ with gestational age have not been consistent [20,45]. In our data, we only found TNF-R2 increased with gestational age, although the range of gestational age in our sample was restricted (9 to 33 weeks). Both among women contemplating pregnancy and pregnant participants, we found obesity associated with CRP and IL-6, which is consistent with previous findings [31,35,36,49]. We found an association between obesity and TNF-R2 among participants contemplating pregnancy but not among pregnant participants. A prior study of pregnant women similarly found no association of obesity with TNF-R2 [31].

Our study has important limitations. First, most women enrolled were non-Hispanic White (90.6%) and had high educational attainment: only 1 participant had less than a bachelor’s degree, and 14.1% had doctoral degrees. Exposure to childhood maltreatment may lead to lower educational attainment [51,52]. Thus, by selecting a sample of professional women we may have excluded women who were most negatively affected by abuse. Consistent with this possibility, many women in our sample had never experienced any of the maltreatment items queried (contemplating pregnancy: 13.7%; pregnant women: 17.7%). This sociodemographic homogeneity likely results in a restricted range of both exposure severity and biological vulnerability, which could blunt the observed associations and contribute to null findings. Additionally, as our sample is comprised of nurses or nursing students, participants are likely healthier and have better access to health resources compared to the general population, further reducing the likelihood of observing strong biological embedding of adversity. These factors may lead to underestimation of associations and limit the generalizability of our findings to more socioeconomically and racially diverse populations. Second, as with most previous studies, childhood maltreatment was retrospectively ascertained. The CTQ score has good criterion-related validity for childhood maltreatment [39]; however, it is subject to recall bias. Third, although most biomarkers demonstrated acceptable assay precision (intra-assay CVs of 4.3% for hsCRP, 8.6% for IL-6, and 4.6% for TNF-R2), the high intra-assay variability of IFN-γ measurement (CV = 20.1%) may introduce random measurement error, which may potentially attenuate true associations and reduce the precision of our estimates. This variability underscores the importance of cautious interpretation of findings related to IFN-γ and highlights the need for future studies using improved measurement techniques to confirm these results. Fourth, our analysis was limited to four inflammatory biomarkers, which may not fully capture the complexity of immune responses associated with maternal childhood maltreatment. Future studies should consider expanding the biomarkers assessed by including additional candidate markers to better detect immune alterations in this context.

Our study also has notable strengths. Participants have been followed up repeatedly, and a variety of demographic characteristics and potential confounders have been assessed. Our measure of childhood maltreatment is well-validated, in widespread use, and extensive. For each inflammatory biomarker in our study, single-assay methods were used, which can be more accurate and sensitive than multiplex platforms [53].

## Conclusions

We did not observe statistically significant associations between childhood maltreatment and four inflammatory biomarkers, including CRP, IL-6, TNF-R2 and IFN-γ, both among women contemplating pregnancy and among pregnant women. These results should be interpreted with caution given the limited statistical power and potential measurement variability. Further studies among more vulnerable populations are needed to determine whether our findings using a socioeconomically advantaged sample are generalizable. In addition, other biological pathways connecting maternal childhood maltreatment with offspring neurodevelopmental disorders should be investigated.

## Supporting information

S1 File STables (S1-S14 Tables). (DOCX)

S2 File S1 Fig. Biomarker concentrations by severity levels of childhood maltreatment among participants contemplating pregnancy (n = 204), Nurses’ Health Study 3. (TIF)

S3 File S2 Fig. Biomarker concentrations by severity levels of childhood maltreatment among pregnant participants (n = 124), Nurses’ Health Study 3. (TIF)
