# Supplementary material for: Estimating the associations between women’s maltreatment in childhood and inflammatory biomarker levels prior to and during pregnancy
Source: PLoS One. 2025 Sep 8;20(9):e0331905. doi: 10.1371/journal.pone.0331905 (PMC12416833; doi:10.1371/journal.pone.0331905)
Supplement: S1 File — (DOCX) [file pone.0331905.s001.docx]

**Supplementary Files**

**S1 Table. Characteristics of study participants contemplating pregnancy by blood collection kit returned or not, Nurses’ Health Study 3, n=583.**

| **Variable** | | **Returned blood kit (n=204)** | | **Did not return blood kit (n=379)** | | **Total (n=583)** | |
| --- | --- | --- | --- | --- | --- | --- | --- |
|  |  | **N or median** | **% or Q1-Q3** | **N or median** | **% or Q1-Q3** | **N or median** | **% or Q1-Q3** |
| **Age** | Age entered NHS3 | 26.5 | 24.5-29.3 | 27.0 | 24.4-30.0 | 26.8 | 24.4-29.8 |
| **Race** | White | 180 | 88.2 | 339 | 91.9 | 519 | 90.6 |
|  | Non-White | 24 | 11.8 | 30 | 8.1 | 54 | 9.4 |
| **Ethnicity** | Hispanic | 12 | 6.0 | 24 | 6.7 | 36 | 6.5 |
|  | Non-Hispanic | 189 | 94.0 | 333 | 93.3 | 522 | 93.6 |
| **Education** | Under bachelor's degree | 0 | 0.0 | 4 | 1.1 | 4 | 0.7 |
|  | Bachelor's degree or RN, Registered Nurse | 81 | 39.7 | 155 | 42.0 | 236 | 41.2 |
|  | Master’s degree | 99 | 48.5 | 155 | 42.0 | 254 | 44.3 |
|  | Doctoral degree | 24 | 11.8 | 55 | 14.9 | 79 | 13.8 |
| **BMI** | Underweight | 6 | 2.9 | 3 | 1.0 | 9 | 1.8 |
|  | Healthy weight | 101 | 49.5 | 133 | 42.9 | 234 | 45.5 |
|  | Overweight | 62 | 30.4 | 90 | 29.0 | 152 | 29.6 |
|  | Obese | 35 | 17.2 | 84 | 27.1 | 119 | 23.1 |
| **Smoking** | Never | 172 | 84.3 | 310 | 84.0 | 482 | 84.1 |
|  | Ever smoked | 32 | 15.7 | 59 | 16.0 | 91 | 15.9 |
| **E-cigarette** | Never | 190 | 93.1 | 339 | 91.9 | 529 | 92.3 |
|  | Ever used | 14 | 6.9 | 30 | 8.1 | 44 | 7.7 |
| **CBD** | Never | 184 | 90.2 | 366 | 99.2 | 550 | 96.0 |
|  | Ever used | 20 | 9.8 | 3 | 0.8 | 23 | 4.0 |
| **Marijuana** | Never | 74 | 36.3 | 148 | 40.1 | 222 | 38.7 |
|  | Ever used | 130 | 63.7 | 221 | 59.9 | 351 | 58.6 |
| **Medication** | Antidepressants | 49 | 24 | 2 | 0.5 | 51 | 8.9 |
|  | Antibiotics | 7 | 3.4 | 2 | 0.5 | 9 | 1.6 |
|  | Aspirin products | 9 | 4.4 | 5 | 1.4 | 14 | 2.4 |
|  | DHEA | 1 | 0.5 | 1 | 0.3 | 2 | 0.4 |
|  | Estrogen for fertility | 7 | 3.4 | 1 | 0.3 | 8 | 1.4 |
|  | Hormonal contraception | 30 | 14.7 | 0 | 0.0 | 30 | 5.2 |
|  | Ibuprofen | 21 | 10.3 | 1 | 0.3 | 22 | 3.8 |
|  | Thyroid medications | 23 | 11.3 | 3 | 0.8 | 26 | 4.5 |

a. Missing values of participants contemplating pregnancy who did not return blood collection kit: race n=10 (2.6%), ethnicity n=22 (5.8%), education n=10 (2.6%), BMI n=69 (18.2%), illness record n=10 (2.6%), smoking n=10 (2.6%), e-cigarette n=10 (2.6%), CBD n=10 (2.6%), marijuana n=10 (2.6%), medication n=10 (2.6%).

**S2 Table.** **Characteristics of study pregnant participants by blood collection kit returned or not, Nurses’ Health Study 3, n=320.**

| **Variable** | | **Returned blood kit (n=124)** | | **Did not return blood kit (n=224)** | | **Total (n=320)** | |
| --- | --- | --- | --- | --- | --- | --- | --- |
|  |  | **N or median** | **% or Q1-Q3** | **N or median** | **% or Q1-Q3** | **N or median** | **% or Q1-Q3** |
| **Age** | Age entered NHS3 | 26.2 | 24.3-28.8 | 26.9 | 24.4-28.8 | 26.7 | 24.3-28.9 |
| **Race** | White | 118 | 95.2 | 202 | 93.5 | 294 | 94.2 |
|  | Non-White | 6 | 4.8 | 14 | 6.5 | 18 | 5.8 |
| **Ethnicity** | Hispanic | 9 | 7.3 | 9 | 4.3 | 15 | 4.9 |
|  | Non-Hispanic | 115 | 92.7 | 203 | 95.8 | 293 | 95.1 |
| **Education** | Under bachelor's degree | 1 | 0.8 | 1 | 0.5 | 2 | 0.6 |
|  | Bachelor's degree or RN, Registered Nurse | 42 | 33.9 | 78 | 36.1 | 110 | 35.3 |
|  | Master’s degree | 61 | 49.2 | 108 | 50.0 | 154 | 49.4 |
|  | Doctoral degree | 20 | 16.1 | 29 | 13.4 | 46 | 14.7 |
| **BMI** | Underweight | 3 | 2.4 | 1 | 0.6 | 3 | 1.1 |
|  | Healthy weight | 52 | 41.9 | 92 | 52.6 | 134 | 49.5 |
|  | Overweight | 40 | 32.3 | 46 | 26.3 | 76 | 28.0 |
|  | Obese | 29 | 23.4 | 36 | 20.6 | 58 | 21.4 |
| **Smoking** | Never | 115 | 92.7 | 184 | 85.2 | 272 | 87.2 |
|  | Ever smoked | 9 | 7.3 | 32 | 14.8 | 40 | 12.8 |
| **E-cigarette** | Never | 119 | 96.0 | 195 | 90.3 | 288 | 92.3 |
|  | Ever used | 5 | 4.0 | 21 | 9.7 | 24 | 7.7 |
| **CBD** | Never | 124 | 100 | 212 | 98.2 | 308 | 98.7 |
|  | Ever used | 0 | 0 | 4 | 1.8 | 4 | 1.3 |
| **Marijuana** | Never | 57 | 46 | 84 | 38.9 | 125 | 40.1 |
|  | Ever used | 67 | 54 | 132 | 61.1 | 187 | 59.9 |
| **Medication** | Antidepressants | 14 | 11.3 | 10 | 4.6 | 22 | 7.1 |
|  | Antibiotics | 3 | 2.4 | 1 | 0.5 | 4 | 1.28 |
|  | Aspirin products | 29 | 23.4 | 2 | 0.9 | 23 | 7.4 |
|  | DHEA | 3 | 2.4 | 1 | 0.5 | 3 | 1.0 |
|  | Estrogen for fertility | 1 | 0.8 | 1 | 0.5 | 2 | 0.6 |
|  | Hormonal contraception | 4 | 3.2 | 6 | 2.8 | 6 | 1.9 |
|  | Ibuprofen | 1 | 0.8 | 4 | 1.9 | 5 | 1.6 |
|  | Thyroid medications | 14 | 11.3 | 3 | 1.4 | 15 | 4.8 |

a. Missing value of pregnant participants who did not return blood collection kit: race n=8 (3.6%), ethnicity n=12 (5.4%), education n=8 (3.6%), BMI n=49 (21.9%), illness record n=8 (3.6%), smoking n=8 (3.6%), e-cigarette n=8 (3.6%), CBD n=8 (3.6%), marijuana n=8 (3.6%), medication n=8 (3.6%).

**S3 Table. Relevant questions from the NHS3 data files used for the CTQ score imputation.**

| **CTQ category** | **Relevant questions from the NHS3 data files** | **Options** |
| --- | --- | --- |
| Emotional abuse | (1) When you were a child (before age 11) did any of the following things happen to you? - People in my family said hurtful or insulting things to me.  (2) When you were a child (before age 11) did any of the following things happen to you? - Someone in my family yelled and screamed at me. | 1. Never true  2. Rarely true  3. Sometimes true  4. Often true  5. Very often true |
| Physical abuse | (1) When you were a child (before age 11) did any of the following things happen to you? - People in my family hit me so hard that it left me with bruises and marks.  (2) When you were a child (before age 11) did any of the following things happen to you? - I was punished with a belt, a board, a cord, or some other hard object.  (3) When you were a child (before age 11) did your parent, step-parent, or adult guardian ever: Spank you for discipline  (4) When you were a child (before age 11) did your parent, step-parent, or adult guardian ever: Push, grab or shove you  (5) When you were a child (before age 11) did your parent, step-parent, or adult guardian ever: Kick, bite or punch you  (6) When you were a child (before age 11) did your parent, step-parent, or adult guardian ever: Hit you with something that hurt your body  (7) When you were a child (before age 11) did your parent, step-parent, or adult guardian ever: Choke or burn you  (8) When you were a child (before age 11) did your parent, step-parent, or adult guardian ever: Physically attack you in some other way  (9) When you were a teenager (ages 11-17) did your parent, step-parent, or adult guardian ever: Push, grab or shove you  (10) When you were a teenager (ages 11-17) did your parent, step-parent, or adult guardian ever: Kick, bite or punch you  (11) When you were a teenager (ages 11-17) did your parent, step-parent, or adult guardian ever: Hit you with something that hurt your body  (12) When you were a teenager (ages 11-17) did your parent, step-parent, or adult guardian ever: Choke or burn you  (13) When you were a teenager (ages 11-17) did your parent, step-parent, or adult guardian ever: Physically attack you in some other way | Question (1)-(2):  1. Never true  2. Rarely true  3. Sometimes true  4. Often true  5. Very often true  Question (3)-(13):  1. Never  2. Once  3. A few times  4. More than a few times |
| Sexual abuse | (1) When you were a child (before age 11), were you ever touched in a sexual way by an adult or older child or were you forced to touch an adult or older child in a sexual way when you did not want to?  (2) When you were a teenager (ages 11-17), were you ever touched in a sexual way by an adult or older child or were you forced to touch an adult or older child in a sexual way when you did not want to?  (3) When you were a child (before age 11) did an adult or older child ever force you or attempt to force you into any sexual activity by threatening you, holding you down, or hurting you in some way  (4) When you were a teenager (ages 11-17) did an adult or older child ever force you or attempt to force you into any sexual activity by threatening you, holding you down, or hurting you in some way | 1. No, this never happened  2. Yes, this happened once  3. Yes, this happened more than once |
| Emotional neglect^a^ | (1) When you were a child (before age 11) did any of the following things happen to you? - I felt like there was someone in my family who wanted me to be a success  (2) When you were a child (before age 11) did any of the following things happen to you? - There was someone in my family who helped me feel that I was important or special  (3) When you were a child (before age 11) did any of the following things happen to you? - My family was a source of strength and support | 1. Never true  2. Rarely true  3. Sometimes true  4. Often true  5. Very often true |
| Physical neglect | (1) Before age 18, - Did you often or very often feel that you didnt have enough to eat, had to wear dirty clothes, and had no one to protect you?  (2) Before age 18,: Were your parents too drunk or high to take care of you or take you to the doctor if you needed it? | 1. Yes  2. No |

NHS3, Nurses’ Health Study 3; CTQ, childhood trauma questionnaire

^a^ Labelled question scores were reversed in the final summed total score.

**S4 Table. Eigenvalues and variance explained from principal component analysis of four inflammatory biomarkers**

| **Component** | **Eigenvalue** | **Difference** | **Proportion of Variance** | **Cumulative Proportion** |
| --- | --- | --- | --- | --- |
| 1 | 1.819 | 0.948 | 0.4548 | 0.4548 |
| 2 | 0.872 | 0.126 | 0.2179 | 0.6727 |
| 3 | 0.745 | 0.182 | 0.1864 | 0.8591 |
| 4 | 0.564 | – | 0.1409 | 1.0000 |

a. Four inflammatory biomarkers: HsCRP, high-sensitive C-reactive protein. IL-6, interleukin-6. TNF-R2, tumor necrosis factor-alpha receptor 2. IFN-γ, interferon-γ.

**S5 Table. Full model results for associations between childhood maltreatment and CRP concentrations among participants contemplating pregnancy, N=204, Nurses’ Health Study 3.**

| **Model** | **Covariate** | **CTQ as a categorical variable**  **(ref: no maltreatment)** | | | | **CTQ as a continuous variable** | | | |
| --- | --- | --- | --- | --- | --- | --- | --- | --- | --- |
|  |  | **β estimate** | **Lower CL** | **Upper CL** | **P-value** | **β estimate** | **Lower CL** | **Upper CL** | **P-value** |
| **Model 1** | Very low maltreatment | -0.2162 | -0.8184 | 0.3860 | 0.4816 | - | - | - | - |
|  | Low maltreatment | 0.3680 | -0.2546 | 0.9907 | 0.2466 | - | - | - | - |
|  | Moderate or severe maltreatment | -0.3565 | -0.9936 | 0.2806 | 0.2728 | - | - | - | - |
|  | Continuous CTQ score | - | - | - | - | -0.0084 | -0.0219 | 0.0051 | 0.2248 |
|  | Age | 0.0406 | -0.0044 | 0.0856 | 0.0772 | 0.0362 | -0.0097 | 0.0820 | 0.1220 |
|  | Hours after waking up | -0.0784 | -0.1570 | 0.0001 | 0.0504 | -0.0795 | -0.1622 | 0.0033 | 0.0598 |
|  | Hours since last eat or drink | -0.0210 | -0.0662 | 0.0241 | 0.3615 | -0.0131 | -0.0588 | 0.0325 | 0.5726 |
|  | Non-White vs White | -0.3444 | -0.8620 | 0.1733 | 0.1923 | -0.2927 | -0.7991 | 0.2137 | 0.2573 |
|  |  |  |  |  |  |  |  |  |  |
| **Model 2** | Very low maltreatment | -0.2133 | -0.7307 | 0.3040 | 0.4190 | - | - | - | - |
|  | Low maltreatment | 0.1304 | -0.4405 | 0.7014 | 0.6544 | - | - | - | - |
|  | Moderate or severe maltreatment | -0.4675 | -0.9856 | 0.0507 | 0.0770 | - | - | - | - |
|  | Continuous CTQ score | - | - | - | - | -0.0084 | -0.0193 | 0.0026 | 0.1357 |
|  | Age | **0.0436** | **0.0046** | **0.0826** | **0.0286** | 0.0394 | -0.0005 | 0.0794 | 0.0530 |
|  | Non-White vs White | -0.3271 | -0.7698 | 0.1156 | 0.1476 | -0.3061 | -0.7324 | 0.1202 | 0.1593 |
|  | Hours after waking up | -0.0583 | -0.1326 | 0.0160 | 0.1242 | -0.0549 | -0.1348 | 0.0250 | 0.1779 |
|  | Hours since last eat or drink | -0.0144 | -0.0542 | 0.0254 | 0.4777 | -0.0073 | -0.0486 | 0.0340 | 0.7278 |
|  | BMI - underweight | -0.4915 | -1.3833 | 0.4003 | 0.2801 | -0.3944 | -1.2601 | 0.4712 | 0.3718 |
|  | BMI - overweight | **0.6634** | **0.3174** | **1.0095** | **0.0002** | **0.6473** | **0.2894** | **1.0052** | **0.0004** |
|  | BMI - obesity | **1.5294** | **1.1178** | **1.9410** | **<0.0001** | **1.5723** | **1.1622** | **1.9825** | **<0.0001** |
|  | Education - master’s degree | -0.0506 | -0.3848 | 0.2835 | 0.7664 | -0.0115 | -0.3378 | 0.3149 | 0.9452 |
|  | Education - Dr degree | 0.1162 | -0.3974 | 0.6298 | 0.6575 | 0.2267 | -0.3021 | 0.7554 | 0.4008 |
|  | Ever smoked | **-0.4396** | **-0.8625** | **-0.0166** | **0.0417** | -0.4337 | -0.8808 | 0.0134 | 0.0573 |
|  | Ever used e-cigarette | **-0.6156** | **-1.1279** | **-0.1032** | **0.0185** | **-0.6958** | **-1.2469** | **-0.1446** | **0.0134** |
|  | Ever used marijuana | 0.1477 | -0.1933 | 0.4887 | 0.3959 | 0.1506 | -0.2015 | 0.5027 | 0.4019 |
|  | Currently using antidepressants | 0.0678 | -0.2917 | 0.4273 | 0.7116 | 0.0580 | -0.3041 | 0.4201 | 0.7535 |
|  | Currently using hormonal contraception | **0.6322** | **0.1503** | **1.1141** | **0.0101** | **0.6334** | **0.1589** | **1.1079** | **0.0089** |
|  | Currently using ibuprofen | 0.4443 | -0.0776 | 0.9663 | 0.0952 | 0.4245 | -0.1357 | 0.9847 | 0.1375 |
|  | Currently using thyroid medications | 0.4067 | -0.0514 | 0.8647 | 0.0819 | 0.4205 | -0.0401 | 0.8811 | 0.0736 |

CRP, C-reactive protein. CL, confidence level. CTQ, childhood trauma questionnaire. Ref, reference group.

a. Reference group: BMI - healthy weight, education - bachelor's degree.

b. Bold font indicates significance (P-value < 0.05).

**S6 Table. Full model results for associations between childhood maltreatment and IL-6 concentrations among participants contemplating pregnancy, N=204, Nurses’ Health Study 3.**

| **Model** | **Covariate** | **CTQ as a categorical variable**  **(ref: no maltreatment)** | | | | **CTQ as a continuous variable** | | | |
| --- | --- | --- | --- | --- | --- | --- | --- | --- | --- |
|  |  | **β estimate** | **Lower CL** | **Upper CL** | **P-value** | **β estimate** | **Lower CL** | **Upper CL** | **P-value** |
| **Model 1** | Very low maltreatment | 0.0319 | -0.1156 | 0.1794 | 0.6718 | - | - | - | - |
|  | Low maltreatment | -0.1044 | -0.2504 | 0.0416 | 0.1612 | - | - | - | - |
|  | Moderate or severe maltreatment | -0.0320 | -0.1820 | 0.1180 | 0.6758 | - | - | - | - |
|  | Continuous CTQ score | - | - | - | - | -0.0022 | -0.0056 | 0.0012 | 0.2003 |
|  | Age | -0.0013 | -0.0126 | 0.0100 | 0.8193 | -0.0007 | -0.0120 | 0.0107 | 0.9061 |
|  | Hours after waking up | -0.0082 | -0.0347 | 0.0182 | 0.5409 | -0.0059 | -0.0325 | 0.0208 | 0.6663 |
|  | Hours since last eat or drink | -0.0019 | -0.0145 | 0.0107 | 0.7685 | -0.0028 | -0.0157 | 0.0102 | 0.6739 |
|  | Non-White vs White | 0.1284 | -0.0164 | 0.2732 | 0.0823 | 0.1318 | -0.0175 | 0.2812 | 0.0837 |
|  |  |  |  |  |  |  |  |  |  |
| **Model 2** | Very low maltreatment | 0.0724 | -0.0722 | 0.2169 | 0.3264 | - | - | - | - |
|  | Low maltreatment | -0.0303 | -0.1675 | 0.1069 | 0.6650 | - | - | - | - |
|  | Moderate or severe maltreatment | 0.0208 | -0.1215 | 0.1631 | 0.7743 | - | - | - | - |
|  | Continuous CTQ score | - | - | - | - | -0.0021 | -0.0055 | 0.0013 | 0.2248 |
|  | Age | 0.0013 | -0.0093 | 0.0120 | 0.8072 | 0.0017 | -0.0088 | 0.0122 | 0.7550 |
|  | Non-White vs White | 0.0694 | -0.0229 | 0.2490 | 0.1030 | 0.1255 | -0.0159 | 0.2669 | 0.0820 |
|  | Hours after waking up | -0.0144 | -0.0401 | 0.0114 | 0.2742 | -0.0128 | -0.0388 | 0.0132 | 0.3340 |
|  | Hours since last eat or drink | -0.0030 | -0.0155 | 0.0094 | 0.6316 | -0.0042 | -0.0170 | 0.0086 | 0.5206 |
|  | BMI - underweight | 0.1361 | -0.0931 | 0.3652 | 0.2445 | 0.1291 | -0.0676 | 0.3257 | 0.1983 |
|  | BMI - overweight | -0.0980 | -0.2047 | 0.0086 | 0.0717 | -0.0937 | -0.2010 | 0.0135 | 0.0868 |
|  | BMI - obesity | **-0.3522** | **-0.4837** | **-0.2208** | **<0.0001** | **-0.3682** | **-0.5015** | **-0.2349** | **<0.0001** |
|  | Education - master’s degree | 0.0529 | -0.0509 | 0.1566 | 0.3180 | 0.0423 | -0.0599 | 0.1444 | 0.4175 |
|  | Education - Dr degree | 0.0710 | -0.0815 | 0.2235 | 0.3616 | 0.0485 | -0.0991 | 0.1960 | 0.5197 |
|  | Ever smoked | 0.0316 | -0.1023 | 0.1656 | 0.6435 | 0.0394 | -0.0963 | 0.1750 | 0.5694 |
|  | Ever used e-cigarette | 0.0513 | -0.1529 | 0.2556 | 0.6222 | 0.0520 | -0.1519 | 0.2559 | 0.6173 |
|  | Ever used marijuana | 0.0050 | -0.0998 | 0.1099 | 0.9250 | -0.0007 | -0.1076 | 0.1062 | 0.9897 |
|  | Currently using antidepressants | -0.0656 | -0.1714 | 0.0401 | 0.2239 | -0.0632 | -0.1686 | 0.0422 | 0.2400 |
|  | Currently using hormonal contraception | 0.0533 | -0.0682 | 0.1748 | 0.3896 | 0.0512 | -0.0709 | 0.1734 | 0.4110 |
|  | Currently using ibuprofen | 0.0342 | -0.1275 | 0.1958 | 0.6787 | 0.0277 | -0.1347 | 0.1902 | 0.7379 |
|  | Currently using thyroid medications | -0.0753 | -0.2277 | 0.0771 | 0.3329 | -0.0639 | -0.2169 | 0.0891 | 0.4129 |

IL-6, interleukin-6. CL, confidence level. CTQ, childhood trauma questionnaire. Ref, reference group.

a. Reference group: BMI - healthy weight, education - bachelor's degree.

b. Bold font indicates significance (P-value < 0.05).

**S7 Table. Full model results for associations between childhood maltreatment and TNF-R2 concentrations among participants contemplating pregnancy, N=204, Nurses’ Health Study 3.**

| **Model** | **Covariate** | **CTQ as a categorical variable**  **(ref: no maltreatment)** | | | | **CTQ as a continuous variable** | | | |
| --- | --- | --- | --- | --- | --- | --- | --- | --- | --- |
|  |  | **β estimate** | **Lower CL** | **Upper CL** | **P-value** | **β estimate** | **Lower CL** | **Upper CL** | **P-value** |
| **Model 1** | Very low maltreatment | 3.5010×10^-5^ | -1.2960×10^-5^ | 8.2990×10^-5^ | 0.1526 | - | - | - | - |
|  | Low maltreatment | -2.7100×10^-6^ | -4.9210×10^-5^ | 4.3790×10^-5^ | 0.9090 | - | - | - | - |
|  | Moderate or severe maltreatment | 2.3780×10^-5^ | -2.5130×10^-5^ | 7.2690×10^-5^ | 0.3407 | - | - | - | - |
|  | Continuous CTQ score | - | - | - | - | 7.4255×10^-7^ | -4.6502×10^-7^ | 1.9500×10^-6^ | 0.2281 |
|  | Age | 2.2180×10^-5^ | -1.9210×10^-5^ | 6.3570×10^-5^ | 0.2936 | 2.1450×10^-5^ | -1.9170×10^-5^ | 6.2070×10^-5^ | 0.3006 |
|  | Age*age | -3.0704×10^-7^ | -8.7837×10^-7^ | 2.6430×10^-7^ | 0.2922 | -2.9671×10^-7^ | -8.5748×10^-7^ | 2.6405×10^-7^ | 0.2997 |
|  | Hours after waking up | **1.2220×10^-5^** | **4.8400×10^-6^** | **1.9600×10^-5^** | **0.0012** | **1.2530×10^-5^** | **5.0500×10^-6^** | **2.0010×10^-5^** | **0.0010** |
|  | Hours since last eat or drink | 1.3700×10^-6^ | -2.2700×10^-6^ | 5.0200×10^-6^ | 0.4597 | 8.1712×10^-7^ | -2.7800×10^-6^ | 4.4100×10^-6^ | 0.6560 |
|  | Non-White vs White | 3.8530×10^-5^ | -9.7300×10^-6^ | 8.6790×10^-5^ | 0.1177 | 3.4870×10^-5^ | -1.2820×10^-5^ | 8.2570×10^-5^ | 0.1518 |
|  |  |  |  |  |  |  |  |  |  |
| **Model 2** | Very low maltreatment | 4.3730×10^-5^ | -1.3400×10^-6^ | 8.8810×10^-5^ | 0.0572 | - | - | - | - |
|  | Low maltreatment | 1.1210×10^-5^ | -3.1870×10^-5^ | 5.4300×10^-5^ | 0.6100 | - | - | - | - |
|  | Moderate or severe maltreatment | 3.3770×10^-5^ | -1.2490×10^-5^ | 8.0040×10^-5^ | 0.1525 | - | - | - | - |
|  | Continuous CTQ score | - | - | - | - | 8.7306×10^-7^ | -2.1855×10^-7^ | 1.9600×10^-6^ | 0.1170 |
|  | Age | 2.1550×10^-5^ | -1.5870×10^-5^ | 5.8980×10^-5^ | 0.2590 | 2.1400×10^-5^ | -1.5490×10^-5^ | 5.8290×10^-5^ | 0.2555 |
|  | Age*age | -2.8911×10^-7^ | -8.0500×10^-7^ | 2.2679×10^-7^ | 0.2720 | -2.8654×10^-7^ | -7.9544×10^-7^ | 2.2236×10^-7^ | 0.2698 |
|  | Non-White vs White | 3.4200×10^-5^ | -1.1300×10^-5^ | 7.9710×10^-5^ | 0.1407 | 3.1050×10^-5^ | -1.3570×10^-5^ | 7.5660×10^-5^ | 0.1726 |
|  | Hours after waking up | **1.1430×10^-5^** | **4.2800×10^-6^** | **1.8580×10^-5^** | **0.0017** | **1.1520×10^-5^** | **4.1500×10^-6^** | **1.8890×10^-5^** | **0.0022** |
|  | Hours since last eat or drink | 1.2400×10^-6^ | -2.3100×10^-6^ | 4.7900×10^-6^ | 0.4934 | 6.4356×10^-7^ | -2.8300×10^-6^ | 4.1200×10^-6^ | 0.7169 |
|  | BMI - underweight | 5.8950×10^-5^ | -4.1960×10^-5^ | 1.5986×10^-4^ | 0.2522 | 4.5630×10^-5^ | -4.3950×10^-5^ | 1.3522×10^-4^ | 0.3181 |
|  | BMI - overweight | -1.0900×10^-5^ | -4.3510×10^-5^ | 2.1710×10^-5^ | 0.5123 | -1.4260×10^-5^ | -4.6750×10^-5^ | 1.8230×10^-5^ | 0.3897 |
|  | BMI - obesity | **-4.9370×10^-5^** | **-8.2980×10^-5^** | **-1.5770×10^-5^** | **0.0040** | **-5.1840×10^-5^** | **-8.6290×10^-5^** | **-1.7400×10^-5^** | **0.0032** |
|  | Education - master’s degree | 1.5300×10^-6^ | -2.5570×10^-5^ | 2.8640×10^-5^ | 0.9117 | -4.1915×10^-7^ | -2.7710×10^-5^ | 2.6870×10^-5^ | 0.9760 |
|  | Education - Dr degree | -6.6500×10^-6^ | -5.6490×10^-5^ | 4.3200×10^-5^ | 0.7938 | -1.6430×10^-5^ | -6.8470×10^-5^ | 3.5600×10^-5^ | 0.5359 |
|  | Ever smoked | 1.9230×10^-5^ | -1.9830×10^-5^ | 5.8290×10^-5^ | 0.3346 | 1.9160×10^-5^ | -2.2080×10^-5^ | 6.0400×10^-5^ | 0.3625 |
|  | Ever used e-cigarette | -2.4360×10^-5^ | -7.2250×10^-5^ | 2.3540×10^-5^ | 0.3189 | -2.4750×10^-5^ | -7.6280×10^-5^ | 2.6770×10^-5^ | 0.3463 |
|  | Ever used marijuana | **3.4490×10^-5^** | **5.2500×10^-6^** | **6.3720×10^-5^** | **0.0208** | **3.3510×10^-5^** | **3.8200×10^-6^** | **6.3210×10^-5^** | **0.0270** |
|  | Currently using antidepressants | 5.4500×10^-6^ | -2.5850×10^-5^ | 3.6740×10^-5^ | 0.7329 | 5.7100×10^-6^ | -2.5370×10^-5^ | 3.6790×10^-5^ | 0.7188 |
|  | Currently using hormonal contraception | 4.0280×10^-5^ | -1.9400×10^-6^ | 8.2500×10^-5^ | 0.0615 | 3.9900×10^-5^ | -1.8100×10^-6^ | 8.1620×10^-5^ | 0.0608 |
|  | Currently using ibuprofen | -6.4800×10^-6^ | -4.9640×10^-5^ | 3.6670×10^-5^ | 0.7684 | -8.1600×10^-6^ | -5.1620×10^-5^ | 3.5290×10^-5^ | 0.7127 |
|  | Currently using thyroid medications | **-4.8300×10^-5^** | **-9.1460×10^-5^** | **-5.1400×10^-6^** | **0.0283** | **-5.2120×10^-5^** | **-9.5600×10^-5^** | **-8.6500×10^-6^** | **0.0188** |

TNF-R2, tumor necrosis factor-alpha receptor 2. CL, confidence level. CTQ, childhood trauma questionnaire. Ref, reference group.

a. Reference group: BMI - healthy weight, education - bachelor's degree.

b. Bold font indicates significance (P-value < 0.05).

**S8 Table. Full model results for associations between childhood maltreatment and IFN-γ concentrations among participants contemplating pregnancy, N=188, Nurses’ Health Study 3.**

| **Model** | **Covariate** | **CTQ as a categorical variable (ref: no maltreatment)** | | | | **CTQ as a continuous variable** | | | |
| --- | --- | --- | --- | --- | --- | --- | --- | --- | --- |
|  |  | **β estimate** | **Lower CL** | **Upper CL** | **P-value** | **β estimate** | **Lower CL** | **Upper CL** | **P-value** |
| **Model 1** | Very low maltreatment | -0.0248 | -0.1454 | 0.0958 | 0.6870 | - | - | - | - |
|  | Low maltreatment | 0.0322 | -0.0909 | 0.1553 | 0.6084 | - | - | - | - |
|  | Moderate or severe maltreatment | 0.0832 | -0.0336 | 0.1999 | 0.1627 | - | - | - | - |
|  | Continuous CTQ score | - | - | - | - | 0.0026 | -0.0002 | 0.0054 | 0.0648 |
|  | Age | -0.0396 | -0.1415 | 0.0622 | 0.4457 | -0.0445 | -0.1442 | 0.0553 | 0.3824 |
|  | Age*age | 0.0006 | -0.0007 | 0.0020 | 0.3674 | 0.0007 | -0.0006 | 0.0020 | 0.3049 |
|  | Hours after waking up | 0.0165 | -0.0061 | 0.0390 | 0.1529 | 0.0136 | -0.0090 | 0.0362 | 0.2375 |
|  | Hours since last eat or drink | 0.0076 | -0.0040 | 0.0192 | 0.1993 | 0.0075 | -0.0041 | 0.0191 | 0.2034 |
|  | Non-White vs White | -0.0327 | -0.1651 | 0.0997 | 0.6283 | -0.0420 | -0.1734 | 0.0893 | 0.5305 |
|  |  |  |  |  |  |  |  |  |  |
| **Model 2** | Very low maltreatment | -0.0210 | -0.1383 | 0.0963 | 0.7252 | - | - | - | - |
|  | Low maltreatment | 0.0392 | -0.0827 | 0.1610 | 0.5288 | - | - | - | - |
|  | Moderate or severe maltreatment | 0.0901 | -0.0301 | 0.2102 | 0.1418 | - | - | - | - |
|  | Continuous CTQ score | - | - | - | - | **0.0030** | **0.0002** | **0.0059** | **0.0365** |
|  | Age | -0.0527 | -0.1484 | 0.0430 | 0.2804 | -0.0579 | -0.1520 | 0.0362 | 0.2275 |
|  | Age*age | 0.0008 | -0.0005 | 0.0021 | 0.2163 | 0.0009 | -0.0004 | 0.0021 | 0.1679 |
|  | Non-White vs White | -0.0553 | -0.1856 | 0.0749 | 0.4051 | -0.0661 | -0.1956 | 0.0634 | 0.3172 |
|  | Hours after wake up | 0.0140 | -0.0091 | 0.0372 | 0.2344 | 0.0117 | -0.0114 | 0.0348 | 0.3212 |
|  | Hours since last eat or drink | 0.0055 | -0.0064 | 0.0174 | 0.3659 | 0.0054 | -0.0065 | 0.0173 | 0.3697 |
|  | BMI - underweight | -0.0122 | -0.1770 | 0.1527 | 0.8848 | -0.0185 | -0.1905 | 0.1536 | 0.8333 |
|  | BMI - overweight | 0.0547 | -0.0412 | 0.1506 | 0.2636 | 0.0565 | -0.0375 | 0.1505 | 0.2387 |
|  | BMI - obesity | -0.0227 | -0.1535 | 0.1082 | 0.7341 | -0.0102 | -0.1401 | 0.1197 | 0.8772 |
|  | Education - master’s degree | 0.0709 | -0.0263 | 0.1681 | 0.1529 | 0.0786 | -0.0185 | 0.1757 | 0.1125 |
|  | Education - Dr degree | 0.0404 | -0.0897 | 0.1706 | 0.5427 | 0.0440 | -0.0800 | 0.1679 | 0.4870 |
|  | Ever smoked | 0.0635 | -0.0747 | 0.2018 | 0.3677 | 0.0602 | -0.0782 | 0.1986 | 0.3939 |
|  | Ever used e-cigarette | 0.0382 | -0.0822 | 0.1585 | 0.5341 | 0.0486 | -0.0765 | 0.1737 | 0.4465 |
|  | Ever used marijuana | **-0.0957** | **-0.1895** | **-0.0019** | **0.0454** | -0.0909 | -0.1858 | 0.0040 | 0.0604 |
|  | Currently using antidepressants | 0.0158 | -0.0798 | 0.1114 | 0.7459 | 0.0209 | -0.0736 | 0.1155 | 0.6644 |
|  | Currently using hormonal contraception | 0.0555 | -0.0698 | 0.1807 | 0.3854 | 0.0519 | -0.0698 | 0.1736 | 0.4030 |
|  | Currently using ibuprofen | -0.0453 | -0.1806 | 0.0900 | 0.5118 | -0.0302 | -0.1668 | 0.1064 | 0.6647 |
|  | Currently using thyroid medications | **-0.1622** | **-0.3093** | **-0.0151** | **0.0307** | **-0.1728** | **-0.3181** | **-0.0276** | **0.0197** |

IFN-γ, interferon-γ. CL, confidence level. CTQ, childhood trauma questionnaire. Ref, reference group.

a. Reference group: BMI - healthy weight, education - bachelor's degree.

b. Bold font indicates significance (P-value < 0.05).

**S9 Table. Full model results for associations between childhood maltreatment and the first principal component of the four inflammatory biomarkers among participants contemplating pregnancy, N=204, Nurses’ Health Study 3.**

| **Model** | **Covariate** | **CTQ as a categorical variable**  **(ref: no maltreatment)** | | | | **CTQ as a continuous variable** | | | |
| --- | --- | --- | --- | --- | --- | --- | --- | --- | --- |
|  |  | **β estimate** | **Lower CL** | **Upper CL** | **P-value** | **β estimate** | **Lower CL** | **Upper CL** | **P-value** |
| **Model 1** | Very low maltreatment | -0.2042 | -0.6445 | 0.2361 | 0.3634 | - | - | - | - |
|  | Low maltreatment | 0.2148 | -0.2427 | 0.6722 | 0.3575 | - | - | - | - |
|  | Moderate or severe maltreatment | -0.2173 | -0.6746 | 0.2401 | 0.3518 | - | - | - | - |
|  | Continuous CTQ score | - | - | - | - | -0.0044 | -0.0128 | 0.0040 | 0.3022 |
|  | Age | 0.0090 | -0.0244 | 0.0424 | 0.5959 | 0.0066 | -0.0269 | 0.0402 | 0.6979 |
|  | Hours after waking up | **-0.0685** | **-0.1348** | **-0.0023** | **0.0427** | **-0.0709** | **-0.1395** | **-0.0022** | **0.0432** |
|  | Hours since last eat or drink | -0.0165 | -0.0502 | 0.0171 | 0.3353 | -0.0112 | -0.0451 | 0.0228 | 0.5198 |
|  | Non-White vs White | **-0.3642** | **-0.7221** | **-0.0063** | **0.0461** | -0.3358 | -0.7017 | 0.0302 | 0.0721 |
|  |  |  |  |  |  |  |  |  |  |
| **Model 2** | Very low maltreatment | -0.2901 | -0.6801 | 0.0998 | 0.1447 | - | - | - | - |
|  | Low maltreatment | 0.0030 | -0.3943 | 0.4003 | 0.9880 | - | - | - | - |
|  | Moderate or severe maltreatment | -0.3502 | -0.7294 | 0.0290 | 0.0703 | - | - | - | - |
|  | Continuous CTQ score | - | - | - | - | -0.0052 | -0.0134 | 0.0030 | 0.2136 |
|  | Age | 0.0050 | -0.0234 | 0.0334 | 0.7284 | 0.0030 | -0.0258 | 0.0318 | 0.8376 |
|  | Non-White vs White | -0.2944 | -0.6433 | 0.0544 | 0.0981 | -0.2841 | -0.6377 | 0.0695 | 0.1153 |
|  | Hours after waking up | -0.0509 | -0.1134 | 0.0116 | 0.1107 | -0.0508 | -0.1157 | 0.0141 | 0.1248 |
|  | Hours since last eat or drink | -0.0111 | -0.0428 | 0.0206 | 0.4920 | -0.0056 | -0.0378 | 0.0266 | 0.7314 |
|  | BMI - underweight | -0.5018 | -1.2952 | 0.2915 | 0.2151 | -0.4119 | -1.1209 | 0.2971 | 0.2548 |
|  | BMI - overweight | 0.2786 | -0.0027 | 0.5599 | 0.0522 | **0.2838** | **0.0032** | **0.5643** | **0.0474** |
|  | BMI - obesity | **1.0532** | **0.7202** | **1.3862** | **<0.0001** | **1.0829** | **0.7371** | **1.4287** | **<0.0001** |
|  | Education - master’s degree | -0.1402 | -0.3938 | 0.1134 | 0.2787 | -0.1187 | -0.3704 | 0.1329 | 0.3551 |
|  | Education - Dr degree | -0.0945 | -0.5293 | 0.3403 | 0.6701 | -0.0072 | -0.4552 | 0.4407 | 0.9747 |
|  | Ever smoked | -0.2970 | -0.6603 | 0.0663 | 0.1091 | -0.3020 | -0.6839 | 0.0799 | 0.1211 |
|  | Ever used e-cigarette | -0.2103 | -0.5738 | 0.1531 | 0.2567 | -0.2444 | -0.6320 | 0.1432 | 0.2165 |
|  | Ever used marijuana | 0.0017 | -0.2643 | 0.2678 | 0.9898 | 0.0068 | -0.2670 | 0.2806 | 0.9611 |
|  | Currently using antidepressants | 0.0738 | -0.1971 | 0.3446 | 0.5935 | 0.0610 | -0.2113 | 0.3334 | 0.6605 |
|  | Currently using hormonal contraception | -0.0893 | -0.4645 | 0.2860 | 0.6411 | -0.0823 | -0.4502 | 0.2856 | 0.6610 |
|  | Currently using ibuprofen | 0.1395 | -0.3293 | 0.6083 | 0.5596 | 0.1366 | -0.3463 | 0.6195 | 0.5794 |
|  | Currently using thyroid medications | **0.4850** | **0.1094** | **0.8605** | **0.0114** | **0.4984** | **0.1115** | **0.8853** | **0.0116** |

CL, confidence level. CTQ, childhood trauma questionnaire. Ref, reference group.

a. Reference group: BMI - healthy weight, education - bachelor's degree.

b. Bold font indicates significance (P-value < 0.05).

**S10 Table. Full model results for associations between childhood maltreatment and CRP concentrations among pregnant participants, N=124, Nurses’ Health Study 3.**

| **Model** | **Covariate** | **CTQ as a categorical variable**  **(ref: no maltreatment)** | | | | **CTQ as a continuous variable** | | | |
| --- | --- | --- | --- | --- | --- | --- | --- | --- | --- |
|  |  | **β estimate** | **Lower CL** | **Upper CL** | **P-value** | **β estimate** | **Lower CL** | **Upper CL** | **P-value** |
| **Model 1** | Very low maltreatment | 0.2337 | -0.2567 | 0.7241 | 0.3503 | - | - | - | - |
|  | Low maltreatment | 0.2202 | -0.3393 | 0.7797 | 0.4406 | - | - | - | - |
|  | Moderate or severe maltreatment | 0.3632 | -0.1697 | 0.8961 | 0.1816 | - | - | - | - |
|  | Continuous CTQ score | - | - | - | - | 0.0099 | -0.0061 | 0.0258 | 0.2270 |
|  | Age | 0.0447 | -0.0138 | 0.1033 | 0.1344 | 0.0426 | -0.0152 | 0.1004 | 0.1490 |
|  | Gestational age | 0.0048 | -0.0219 | 0.0315 | 0.7250 | 0.0048 | -0.0219 | 0.0315 | 0.7258 |
|  | Hours after waking up | -0.0474 | -0.1455 | 0.0508 | 0.3442 | -0.0541 | -0.1495 | 0.0412 | 0.2659 |
|  | Hours since last eat or drink | 0.0130 | -0.0456 | 0.0715 | 0.6640 | 0.0088 | -0.0502 | 0.0677 | 0.7705 |
|  | Non-White vs White | -0.3932 | -1.0969 | 0.3106 | 0.2735 | -0.3568 | -1.0622 | 0.3487 | 0.3216 |
|  |  |  |  |  |  |  |  |  |  |
| **Model 2** | Very low maltreatment | 0.2573 | -0.1556 | 0.6702 | 0.2219 | - | - | - | - |
|  | Low maltreatment | 0.2593 | -0.2638 | 0.7825 | 0.3312 | - | - | - | - |
|  | Moderate or severe maltreatment | 0.3854 | -0.0959 | 0.8666 | 0.1165 | - | - | - | - |
|  | Continuous CTQ score | - | - | - | - | 0.0136 | -0.0012 | 0.0283 | 0.0709 |
|  | Age | 0.0230 | -0.0372 | 0.0832 | 0.4546 | 0.0223 | -0.0364 | 0.0809 | 0.4571 |
|  | Gestational age | -0.0025 | -0.0253 | 0.0203 | 0.8301 | -0.0026 | -0.0254 | 0.0203 | 0.8262 |
|  | Non-White vs White | -0.3389 | -1.0701 | 0.3923 | 0.3637 | -0.3240 | -1.0792 | 0.4311 | 0.4004 |
|  | Hours after waking up | -0.0393 | -0.1357 | 0.0571 | 0.4246 | -0.0455 | -0.1403 | 0.0492 | 0.3461 |
|  | Hours since last eat or drink | -0.0038 | -0.0622 | 0.0545 | 0.8975 | -0.0081 | -0.0659 | 0.0496 | 0.7821 |
|  | BMI - underweight | -0.1028 | -0.8716 | 0.6660 | 0.7933 | -0.0676 | -0.8606 | 0.7253 | 0.8672 |
|  | BMI - overweight | **0.5289** | **0.1340** | **0.9239** | **0.0087** | **0.5346** | **0.1463** | **0.9229** | **0.0070** |
|  | BMI - obesity | **1.3004** | **0.8845** | **1.7163** | **<0.0001** | **1.2990** | **0.8811** | **1.7169** | **<0.0001** |
|  | Education - under bachelor's degree | -0.7057 | -1.4672 | 0.0559 | 0.0694 | **-0.6843** | **-1.2518** | **-0.1169** | **0.0181** |
|  | Education - master’s degree | 0.1287 | -0.2545 | 0.5120 | 0.5103 | 0.1355 | -0.2269 | 0.4979 | 0.4637 |
|  | Education - Dr degree | -0.0749 | -0.5304 | 0.3806 | 0.7473 | -0.0758 | -0.5295 | 0.3779 | 0.7435 |
|  | Ever smoked | -0.0203 | -0.5307 | 0.4900 | 0.9377 | -0.1319 | -0.6870 | 0.4232 | 0.6415 |
|  | Ever used e-cigarette | -0.2526 | -0.8515 | 0.3462 | 0.4083 | -0.1768 | -0.7900 | 0.4364 | 0.5721 |
|  | Ever used marijuana | 0.0854 | -0.2470 | 0.4179 | 0.6145 | 0.0588 | -0.2665 | 0.3841 | 0.7231 |
|  | Currently using antidepressants | **-0.5090** | **-0.9521** | **-0.0658** | **0.0244** | **-0.4840** | **-0.9134** | **-0.0546** | **0.0272** |
|  | Currently using aspirin products | 0.3152 | -0.0214 | 0.6519 | 0.0665 | 0.3066 | -0.0401 | 0.6533 | 0.0830 |
|  | Currently using thyroid medications | 0.0518 | -0.4331 | 0.5367 | 0.8340 | 0.0761 | -0.4227 | 0.5750 | 0.7649 |

CRP, C-reactive protein. CL, confidence level. CTQ, childhood trauma questionnaire. Ref, reference group.

a. Reference group: BMI - healthy weight, education - bachelor's degree.

b. Bold font indicates significance (P-value < 0.05).

**S11 Table. Full model results for associations between childhood maltreatment and IL-6 concentrations among pregnant participants, N=123, Nurses’ Health Study 3.**

| **Model** | **Covariate** | **CTQ as a categorical variable**  **(ref: no maltreatment)** | | | | **CTQ as a continuous variable** | | | |
| --- | --- | --- | --- | --- | --- | --- | --- | --- | --- |
|  |  | **β estimate** | **Lower CL** | **Upper CL** | **P-value** | **β estimate** | **Lower CL** | **Upper CL** | **P-value** |
| **Model 1** | Very low maltreatment | -0.1236 | -0.3353 | 0.0881 | 0.2523 | - | - | - | - |
|  | Low maltreatment | -0.1115 | -0.2834 | 0.0603 | 0.2034 | - | - | - | - |
|  | Moderate or severe maltreatment | -0.0717 | -0.2689 | 0.1254 | 0.4756 | - | - | - | - |
|  | Continuous CTQ score | - | - | - | - | 0.0009 | -0.0042 | 0.0060 | 0.7313 |
|  | Age | -0.0054 | -0.0256 | 0.0148 | 0.5985 | -0.0034 | -0.0229 | 0.0161 | 0.7324 |
|  | Gestational age | -0.0045 | -0.0137 | 0.0047 | 0.3349 | -0.0049 | -0.0143 | 0.0045 | 0.3062 |
|  | Hours after waking up | 0.0001 | -0.0315 | 0.0316 | 0.9970 | 0.0032 | -0.0264 | 0.0329 | 0.8320 |
|  | Hours since last eat or drink | -0.0110 | -0.0281 | 0.0062 | 0.2099 | -0.0104 | -0.0281 | 0.0073 | 0.2491 |
|  | Non-White vs White | -0.0356 | -0.1939 | 0.1226 | 0.6593 | -0.0526 | -0.2109 | 0.1056 | 0.5145 |
|  |  |  |  |  |  |  |  |  |  |
| **Model 2** | Very low maltreatment | -0.1529 | -0.3640 | 0.0581 | 0.1554 | - | - | - | - |
|  | Low maltreatment | -0.1130 | -0.2843 | 0.0584 | 0.1963 | - | - | - | - |
|  | Moderate or severe maltreatment | -0.0910 | -0.2871 | 0.1050 | 0.3629 | - | - | - | - |
|  | Continuous CTQ score | - | - | - | - | 0.0007 | -0.0042 | 0.0056 | 0.7817 |
|  | Age | 0.0079 | -0.0136 | 0.0293 | 0.4734 | 0.0100 | -0.0111 | 0.0310 | 0.3537 |
|  | Gestational age | -0.0031 | -0.0116 | 0.0054 | 0.4709 | -0.0035 | -0.0121 | 0.0052 | 0.4346 |
|  | Non-White vs White | -0.0996 | -0.2178 | 0.0186 | 0.0987 | -0.1102 | -0.2308 | 0.0105 | 0.0735 |
|  | Hours after waking up | -0.0064 | -0.0406 | 0.0277 | 0.7117 | -0.0021 | -0.0341 | 0.0299 | 0.8979 |
|  | Hours since last eat or drink | -0.0051 | -0.0222 | 0.0119 | 0.5560 | -0.0041 | -0.0214 | 0.0132 | 0.6437 |
|  | BMI - underweight | 0.1000 | -0.3471 | 0.5470 | 0.6612 | 0.0714 | -0.3554 | 0.4982 | 0.7429 |
|  | BMI - overweight | -0.1254 | -0.2768 | 0.0260 | 0.1045 | -0.1214 | -0.2738 | 0.0310 | 0.1185 |
|  | BMI - obesity | **-0.2809** | **-0.4268** | **-0.1350** | **0.0002** | **-0.2795** | **-0.4274** | **-0.1317** | **0.0002** |
|  | Education - under bachelor's degree | 0.0723 | -0.1929 | 0.3375 | 0.5931 | 0.0668 | -0.1672 | 0.3007 | 0.5759 |
|  | Education - master’s degree | -0.0507 | -0.1797 | 0.0783 | 0.4413 | -0.0598 | -0.1850 | 0.0654 | 0.3494 |
|  | Education - Dr degree | -0.1483 | -0.3619 | 0.0652 | 0.1734 | -0.1345 | -0.3488 | 0.0797 | 0.2185 |
|  | Ever smoked | -0.0237 | -0.2225 | 0.1752 | 0.8156 | -0.0318 | -0.2506 | 0.1870 | 0.7756 |
|  | Ever used e-cigarette | 0.0461 | -0.2233 | 0.3156 | 0.7372 | 0.0310 | -0.2572 | 0.3192 | 0.8329 |
|  | Ever used marijuana | -0.1016 | -0.2323 | 0.0290 | 0.1274 | -0.0895 | -0.2201 | 0.0411 | 0.1792 |
|  | Currently using antidepressants | 0.0236 | -0.1525 | 0.1997 | 0.7929 | 0.0040 | -0.1704 | 0.1784 | 0.9640 |
|  | Currently using aspirin products | -0.0697 | -0.2061 | 0.0667 | 0.3166 | -0.0663 | -0.2043 | 0.0718 | 0.3467 |
|  | Currently using thyroid medications | -0.0204 | -0.2004 | 0.1596 | 0.8243 | -0.0178 | -0.1917 | 0.1561 | 0.8411 |

IL-6, interleukin-6. CL, confidence level. CTQ, childhood trauma questionnaire. Ref, reference group.

a. Reference group: BMI - healthy weight, education - bachelor's degree.

b. Bold font indicates significance (P-value < 0.05).

**S12 Table. Full model results for associations between childhood maltreatment and TNF-R2 concentrations among pregnant participants, N=123, Nurses’ Health Study 3.**

| **Model** | **Covariate** | **CTQ as a categorical variable**  **(ref: no maltreatment)** | | | | **CTQ as a continuous variable** | | | |
| --- | --- | --- | --- | --- | --- | --- | --- | --- | --- |
|  |  | **β estimate** | **Lower CL** | **Upper CL** | **P-value** | **β estimate** | **Lower CL** | **Upper CL** | **P-value** |
| **Model 1** | Very low maltreatment | -9.5300×10^-6^ | -5.1980×10^-5^ | 3.2930×10^-5^ | 0.6600 | - | - | - | - |
|  | Low maltreatment | -6.4300×10^-6^ | -4.7610×10^-5^ | 3.4750×10^-5^ | 0.7595 | - | - | - | - |
|  | Moderate or severe maltreatment | -8.2100×10^-6^ | -5.3470×10^-5^ | 3.7050×10^-5^ | 0.7222 | - | - | - | - |
|  | Continuous CTQ score | - | - | - | - | -7.5953×10^-7^ | -2.1800×10^-6^ | 6.5736×10^-7^ | 0.2934 |
|  | Age | 1.4710×10^-5^ | -2.5500×10^-5^ | 5.4920×10^-5^ | 0.4734 | 1.8230×10^-5^ | -2.1060×10^-5^ | 5.7530×10^-5^ | 0.3631 |
|  | Age*age | -2.1818×10^-7^ | -8.0783×10^-7^ | 3.7147×10^-7^ | 0.4683 | -2.6714×10^-7^ | -8.4550×10^-7^ | 3.1121×10^-7^ | 0.3653 |
|  | Gestational age | **-5.0700×10^-6^** | **-7.1100×10^-6^** | **-3.0200×10^-6^** | **<0.0001** | **-5.0700×10^-6^** | **-7.1300×10^-6^** | **-3.0200×10^-6^** | **<0.0001** |
|  | Hours after waking up | -1.4050×10^-5^ | -3.5260×10^-5^ | 7.1600×10^-6^ | 0.1943 | -1.3110×10^-5^ | -3.3540×10^-5^ | 7.3200×10^-6^ | 0.2084 |
|  | Square of hours after waking up | **2.0600×10^-6^** | **4.7294×10^-7^** | **3.6500×10^-6^** | **0.0110** | **1.9800×10^-6^** | **4.6298×10^-7^** | **3.4900×10^-6^** | **0.0105** |
|  | Hours since last eat or drink | **-7.4400×10^-6^** | **-1.1030×10^-5^** | **-3.8500×10^-6^** | **<0.0001** | **-6.8800×10^-6^** | **-1.0420×10^-5^** | **-3.3400×10^-6^** | **0.0001** |
|  | Non-White vs White | -4.3780×10^-5^ | -1.1476×10^-4^ | 2.7200×10^-5^ | 0.2267 | -3.9480×10^-5^ | -1.0858×10^-4^ | 2.9610×10^-5^ | 0.2627 |
|  |  |  |  |  |  |  |  |  |  |
| **Model 2** | Very low maltreatment | -1.5160×10^-5^ | -6.0180×10^-5^ | 2.9860×10^-5^ | 0.5093 | - | - | - | - |
|  | Low maltreatment | -7.6500×10^-6^ | -4.9290×10^-5^ | 3.3990×10^-5^ | 0.7188 | - | - | - | - |
|  | Moderate or severe maltreatment | -1.2700×10^-5^ | -5.9260×10^-5^ | 3.3850×10^-5^ | 0.5928 | - | - | - | - |
|  | Continuous CTQ score | - | - | - | - | -1.0700×10^-6^ | -2.5500×10^-6^ | 4.0500×10^-7^ | 0.1547 |
|  | Age | 1.7520×10^-5^ | -3.2560×10^-5^ | 6.7600×10^-5^ | 0.4930 | 2.0460×10^-5^ | -2.6990×10^-5^ | 6.7900×10^-5^ | 0.3981 |
|  | Age*age | -2.4103×10^-7^ | -9.7156×10^-7^ | 4.8950×10^-7^ | 0.5178 | -2.8329×10^-7^ | -9.7904×10^-7^ | 4.1245×10^-7^ | 0.4248 |
|  | Gestational age | -4.8900×10^-6^ | -7.0100×10^-6^ | -2.7700×10^-6^ | **<0.0001** | -4.9000×10^-6^ | -7.0200×10^-6^ | -2.7700×10^-6^ | **<0.0001** |
|  | Non-White vs White | -6.3340×10^-5^ | -1.3510×10^-4^ | 8.4100×10^-6^ | 0.0836 | -5.6250×10^-5^ | -1.2413×10^-4^ | 1.1630×10^-5^ | 0.1043 |
|  | Hours after waking up | -1.4420×10^-5^ | -3.6470×10^-5^ | 7.6400×10^-6^ | 0.2001 | -1.2890×10^-5^ | -3.3970×10^-5^ | 8.1800×10^-6^ | 0.2305 |
|  | Square of hours after waking up | **2.1000×10^-6^** | **5.3542×10^-7^** | **3.6600×10^-6^** | **0.0085** | **1.9800×10^-6^** | **5.0624×10^-7^** | **3.4500×10^-6^** | **0.0084** |
|  | Hours since last eat or drink | **-7.7000×10^-6^** | **-1.2000×10^-5^** | **-3.4000×10^-6^** | **0.0005** | **-6.9400×10^-6^** | **-1.1080×10^-5^** | **-2.8000×10^-6^** | **0.0010** |
|  | BMI - underweight | -3.5980×10^-5^ | -8.7120×10^-5^ | 1.5160×10^-5^ | 0.1679 | -3.6910×10^-5^ | -8.3510×10^-5^ | 9.7000×10^-6^ | 0.1207 |
|  | BMI - overweight | -8.2900×10^-6^ | -4.5190×10^-5^ | 2.8610×10^-5^ | 0.6597 | -1.0270×10^-5^ | -4.7200×10^-5^ | 2.6660×10^-5^ | 0.5857 |
|  | BMI - obesity | -2.4220×10^-5^ | -5.7860×10^-5^ | 9.4200×10^-6^ | 0.1582 | -2.3860×10^-5^ | -5.6360×10^-5^ | 8.6400×10^-6^ | 0.1502 |
|  | Education - under bachelor's degree | -3.2000×10^-6^ | -5.9270×10^-5^ | 5.2860×10^-5^ | 0.9109 | -7.9030×10^-8^ | -4.8660×10^-5^ | 4.8500×10^-5^ | 0.9975 |
|  | Education - master degree | -1.1290×10^-5^ | -4.4580×10^-5^ | 2.2000×10^-5^ | 0.5063 | -1.3660×10^-5^ | -4.4250×10^-5^ | 1.6930×10^-5^ | 0.3813 |
|  | Education - Dr degree | -1.0840×10^-5^ | -5.6170×10^-5^ | 3.4500×10^-5^ | 0.6395 | -1.4160×10^-5^ | -5.9020×10^-5^ | 3.0700×10^-5^ | 0.5362 |
|  | Ever smoked | 2.1410×10^-5^ | -4.1420×10^-5^ | 8.4240×10^-5^ | 0.5042 | 3.1100×10^-5^ | -2.0320×10^-5^ | 8.2520×10^-5^ | 0.2359 |
|  | Ever used e-cigarette | 8.8200×10^-6^ | -8.0880×10^-5^ | 9.8510×10^-5^ | 0.8472 | 4.2600×10^-6^ | -8.6280×10^-5^ | 9.4800×10^-5^ | 0.9265 |
|  | Ever used marijuana | -2.2200×10^-5^ | -5.5160×10^-5^ | 1.0760×10^-5^ | 0.1869 | -1.7650×10^-5^ | -4.7880×10^-5^ | 1.2580×10^-5^ | 0.2524 |
|  | Currently using antidepressants | 9.0700×10^-6^ | -2.6110×10^-5^ | 4.4260×10^-5^ | 0.6132 | 1.3500×10^-5^ | -1.8940×10^-5^ | 4.5930×10^-5^ | 0.4148 |
|  | Currently using aspirin products | -1.6900×10^-5^ | -4.6190×10^-5^ | 1.2390×10^-5^ | 0.2582 | -1.7250×10^-5^ | -4.6290×10^-5^ | 1.1790×10^-5^ | 0.2445 |
|  | Currently using thyroid medications | 2.3590×10^-5^ | -1.6580×10^-5^ | 6.3760×10^-5^ | 0.2497 | 2.3480×10^-5^ | -1.5280×10^-5^ | 6.2240×10^-5^ | 0.2350 |

TNF-R2, tumor necrosis factor-alpha receptor 2. CL, confidence level. CTQ, childhood trauma questionnaire. Ref, reference group.

a. Reference group: BMI - healthy weight, education - bachelor's degree.

b. Bold font indicates significance (P-value < 0.05).

**S13 Table. Full model results for associations between childhood maltreatment and IFN-γ concentrations among pregnant participants, N=123, Nurses’ Health Study 3.**

| **Model** | **Covariate** | **CTQ as a categorical variable**  **(ref: no maltreatment)** | | | | **CTQ as a continuous variable** | | | |
| --- | --- | --- | --- | --- | --- | --- | --- | --- | --- |
|  |  | **β estimate** | **Lower CL** | **Upper CL** | **P-value** | **β estimate** | **Lower CL** | **Upper CL** | **P-value** |
| **Model 1** | Very low maltreatment | 0.0403 | -0.1116 | 0.1923 | 0.6031 | - | - | - | - |
|  | Low maltreatment | 0.0664 | -0.0754 | 0.2081 | 0.3587 | - | - | - | - |
|  | Moderate or severe maltreatment | 0.0194 | -0.1298 | 0.1686 | 0.7989 | - | - | - | - |
|  | Continuous CTQ score | - | - | - | - | -0.0029 | -0.0095 | 0.0037 | 0.3880 |
|  | Age | 0.0057 | -0.0121 | 0.0235 | 0.5310 | 0.0049 | -0.0119 | 0.0216 | 0.5708 |
|  | Gestational age | -0.0005 | -0.0084 | 0.0074 | 0.9054 | -0.0001 | -0.0077 | 0.0076 | 0.9843 |
|  | Hours after waking up | 0.0044 | -0.0174 | 0.0261 | 0.6932 | 0.0035 | -0.0178 | 0.0248 | 0.7470 |
|  | Hours since last eat or drink | **0.1303** | **0.0671** | **0.1935** | **<0.0001** | **0.1248** | **0.0637** | **0.1858** | **<0.0001** |
|  | Square of hours since last eat or drink | **-0.0112** | **-0.0163** | **-0.0061** | **<0.0001** | **-0.0106** | **-0.0155** | **-0.0057** | **<0.0001** |
|  | Non-White vs White | -0.1597 | -0.3992 | 0.0797 | 0.1910 | -0.1470 | -0.3825 | 0.0886 | 0.2213 |
|  |  |  |  |  |  |  |  |  |  |
| **Model 2** | Very low maltreatment | 0.0018 | -0.1524 | 0.1560 | 0.9822 | - | - | - | - |
|  | Low maltreatment | 0.0428 | -0.1044 | 0.1899 | 0.5689 | - | - | - | - |
|  | Moderate or severe maltreatment | -0.0279 | -0.1695 | 0.1137 | 0.6996 | - | - | - | - |
|  | Continuous CTQ score | - | - | - | - | -0.0017 | -0.0064 | 0.0031 | 0.4909 |
|  | Age | 0.0103 | -0.0081 | 0.0287 | 0.2712 | 0.0095 | -0.0082 | 0.0271 | 0.2920 |
|  | Gestational age | -0.0024 | -0.0104 | 0.0057 | 0.5606 | -0.0021 | -0.0102 | 0.0060 | 0.6103 |
|  | Non-White vs White | -0.1421 | -0.4119 | 0.1276 | 0.3017 | -0.1419 | -0.4172 | 0.1335 | 0.3126 |
|  | Hours after waking up | -0.0033 | -0.0260 | 0.0194 | 0.7769 | -0.0029 | -0.0250 | 0.0191 | 0.7935 |
|  | Hours since last eat or drink | **0.1312** | **0.0689** | **0.1934** | **<0.0001** | **0.1292** | **0.0667** | **0.1917** | **<0.0001** |
|  | Square of hours since last eat or drink | **-0.0111** | **-0.0162** | **-0.0060** | **<0.0001** | **-0.0108** | **-0.0159** | **-0.0057** | **<0.0001** |
|  | BMI - underweight | **0.4500** | **0.1834** | **0.7166** | **0.0009** | **0.4337** | **0.1745** | **0.6930** | **0.0010** |
|  | BMI - overweight | 0.0690 | -0.0563 | 0.1943 | 0.2804 | 0.0602 | -0.0640 | 0.1843 | 0.3422 |
|  | BMI - obesity | 0.0632 | -0.0920 | 0.2184 | 0.4245 | 0.0625 | -0.0928 | 0.2178 | 0.4304 |
|  | Education - under bachelor's degree | **0.2839** | **0.1162** | **0.4517** | **0.0009** | **0.3261** | **0.1825** | **0.4698** | **<0.0001** |
|  | Education - master’s degree | -0.0456 | -0.1519 | 0.0607 | 0.4009 | -0.0403 | -0.1454 | 0.0649 | 0.4527 |
|  | Education - Dr degree | -0.1358 | -0.3081 | 0.0365 | 0.1225 | -0.1331 | -0.3081 | 0.0419 | 0.1359 |
|  | Ever smoked | **-0.3206** | **-0.4835** | **-0.1576** | **0.0001** | **-0.3103** | **-0.4752** | **-0.1454** | **0.0002** |
|  | Ever used e-cigarette | -0.1726 | -0.4146 | 0.0694 | 0.1621 | -0.1780 | -0.4214 | 0.0653 | 0.1516 |
|  | Ever used marijuana | -0.0178 | -0.1288 | 0.0931 | 0.7530 | -0.0174 | -0.1227 | 0.0879 | 0.7455 |
|  | Currently using antidepressants | 0.1108 | -0.0713 | 0.2930 | 0.2331 | 0.1210 | -0.0606 | 0.3026 | 0.1915 |
|  | Currently using aspirin products | 0.0606 | -0.0581 | 0.1793 | 0.3169 | 0.0625 | -0.0582 | 0.1833 | 0.3101 |
|  | Currently using thyroid medications | 0.0496 | -0.0980 | 0.1971 | 0.5103 | 0.0487 | -0.1005 | 0.1980 | 0.5223 |

IFN-γ, interferon-γ. CL, confidence level. CTQ, childhood trauma questionnaire. Ref, reference group.

a. Reference group: BMI - healthy weight, education - bachelor's degree.

b. Bold font indicates significance (P-value < 0.05).

**S14 Table. Full model results for associations between childhood maltreatment and the first principal component of the four inflammatory biomarkers among pregnant participants, N=124, Nurses’ Health Study 3.**

| **Model** | **Covariate** | **CTQ as a categorical variable**  **(ref: no maltreatment)** | | | | **CTQ as a continuous variable** | | | |
| --- | --- | --- | --- | --- | --- | --- | --- | --- | --- |
|  |  | **β estimate** | **Lower CL** | **Upper CL** | **P-value** | **β estimate** | **Lower CL** | **Upper CL** | **P-value** |
| **Model 1** | Very low maltreatment | 0.2118 | -0.2708 | 0.6944 | 0.3896 | - | - | - | - |
|  | Low maltreatment | 0.1775 | -0.2594 | 0.6144 | 0.4258 | - | - | - | - |
|  | Moderate or severe maltreatment | 0.1426 | -0.4073 | 0.6926 | 0.6112 | - | - | - | - |
|  | Continuous CTQ score | - | - | - | - | 0.0049 | -0.0154 | 0.0252 | 0.6340 |
|  | Age | 0.0190 | -0.0356 | 0.0736 | 0.4953 | 0.0155 | -0.0363 | 0.0674 | 0.5575 |
|  | Gestational age | **0.0351** | **0.0092** | **0.0610** | **0.0079** | **0.0354** | **0.0092** | **0.0617** | **0.0081** |
|  | Hours after waking up | -0.0690 | -0.1386 | 0.0005 | 0.0518 | **-0.0726** | **-0.1386** | **-0.0065** | **0.0313** |
|  | Hours since last eat or drink | 0.0370 | -0.0027 | 0.0768 | 0.0677 | 0.0332 | -0.0057 | 0.0722 | 0.0943 |
|  | Non-White vs White | -0.1525 | -0.7794 | 0.4744 | 0.6335 | -0.1387 | -0.7170 | 0.4396 | 0.6383 |
|  |  |  |  |  |  |  |  |  |  |
| **Model 2** | Very low maltreatment | 0.3233 | -0.1346 | 0.7811 | 0.1664 | - | - | - | - |
|  | Low maltreatment | 0.1909 | -0.2268 | 0.6087 | 0.3703 | - | - | - | - |
|  | Moderate or severe maltreatment | 0.2636 | -0.2100 | 0.7372 | 0.2753 | - | - | - | - |
|  | Continuous CTQ score | - | - | - | - | 0.0086 | -0.0087 | 0.0260 | 0.3294 |
|  | Age | -0.0157 | -0.0753 | 0.0438 | 0.6044 | -0.0178 | -0.0747 | 0.0391 | 0.5395 |
|  | Gestational age | **0.0300** | **0.0066** | **0.0534** | **0.0121** | **0.0301** | **0.0062** | **0.0541** | **0.0137** |
|  | Non-White vs White | -0.0714 | -0.7749 | 0.6321 | 0.8424 | -0.0678 | -0.7059 | 0.5703 | 0.8351 |
|  | Hours after waking up | -0.0629 | -0.1437 | 0.0178 | 0.1267 | -0.0702 | -0.1461 | 0.0057 | 0.0699 |
|  | Hours since last eat or drink | 0.0191 | -0.0234 | 0.0616 | 0.3778 | 0.0128 | -0.0250 | 0.0506 | 0.5073 |
|  | BMI - underweight | -0.4540 | -1.3009 | 0.3929 | 0.2934 | -0.3888 | -1.2345 | 0.4570 | 0.3676 |
|  | BMI - overweight | 0.2448 | -0.1874 | 0.6770 | 0.2669 | 0.2672 | -0.1714 | 0.7058 | 0.2325 |
|  | BMI - obesity | **0.8777** | **0.5193** | **1.2362** | **<0.0001** | **0.8736** | **0.5198** | **1.2273** | **<0.0001** |
|  | Education - under bachelor's degree | -0.5041 | -1.1513 | 0.1432 | 0.1269 | **-0.5415** | **-1.0722** | **-0.0108** | **0.0455** |
|  | Education - master’s degree | 0.2089 | -0.1766 | 0.5945 | 0.2882 | 0.2325 | -0.1369 | 0.6019 | 0.2173 |
|  | Education - Dr degree | 0.2584 | -0.2209 | 0.7377 | 0.2907 | 0.2654 | -0.2178 | 0.7487 | 0.2817 |
|  | Ever smoked | 0.0693 | -0.5916 | 0.7303 | 0.8371 | -0.0056 | -0.6336 | 0.6224 | 0.9861 |
|  | Ever used e-cigarette | -0.6705 | -2.1384 | 0.7975 | 0.3707 | -0.6066 | -2.1141 | 0.9009 | 0.4303 |
|  | Ever used marijuana | 0.2245 | -0.1353 | 0.5844 | 0.2213 | 0.1803 | -0.1593 | 0.5199 | 0.2981 |
|  | Currently using antidepressants | -0.2680 | -0.6320 | 0.0960 | 0.1490 | -0.2797 | -0.6212 | 0.0618 | 0.1084 |
|  | Currently using aspirin products | 0.2486 | -0.0746 | 0.5717 | 0.1317 | 0.2442 | -0.0902 | 0.5787 | 0.1524 |
|  | Currently using thyroid medications | -0.0820 | -0.5689 | 0.4049 | 0.7412 | -0.0790 | -0.5516 | 0.3936 | 0.7433 |

CL, confidence level. CTQ, childhood trauma questionnaire. Ref, reference group.

a. Reference group: BMI - healthy weight, education - bachelor's degree.

b. Bold font indicates significance (P-value < 0.05).
