# Supplementary figures and images for: Estimating the associations between women’s maltreatment in childhood and inflammatory biomarker levels prior to and during pregnancy
Source: PLoS One. 2025 Sep 8;20(9):e0331905. doi: 10.1371/journal.pone.0331905 (PMC12416833; doi:10.1371/journal.pone.0331905)

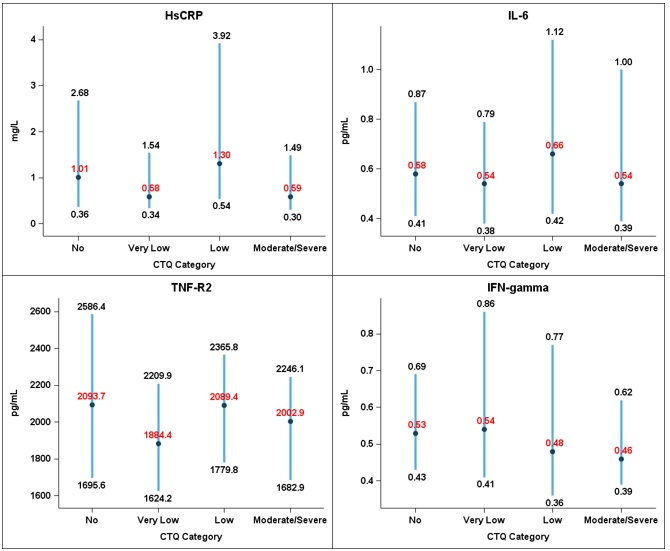

Supplement: S2 File — (TIF) [file pone.0331905.s002.tif]

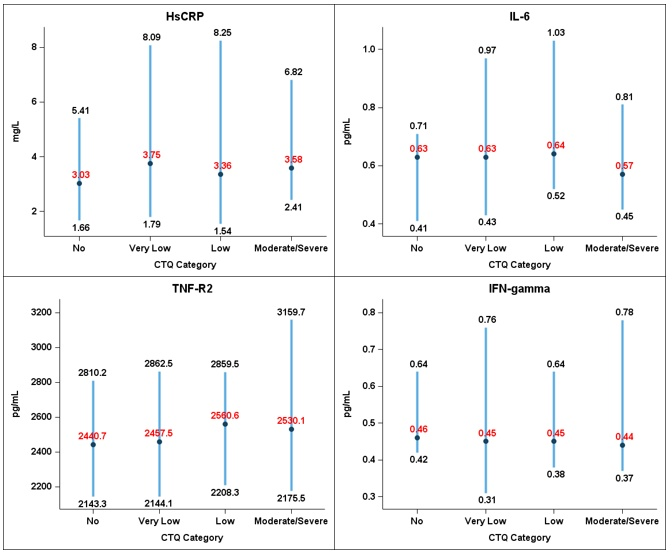

Supplement: S3 File — (TIF) [file pone.0331905.s003.tif]
